# Supplementary material for: Structural insight into small molecule action on Frizzleds
Source: Nat Commun. 2020 Jan 21;11:414. doi: 10.1038/s41467-019-14149-3 (PMC6972889; doi:10.1038/s41467-019-14149-3)
Supplement: Supplementary file 1 — Supplementary Information [file 41467_2019_14149_MOESM1_ESM.pdf]

Supplementary Fig. 1. Sequence alignment of human SMO, FZD_6 and FZD_4. Sequence alignment of the protein sequences of human SMO, FZD_6 and FZD_4 (excluding the CRDs and the C-termini) generated using ClustalX2 software[1] and visualized in UGENE[2]. Red squares indicate the amino acid residues (main chain or side chain) located within 4 Å from SAG1.3 in SMO and FZD_6 throughout the MD simulations (observed after every 200th ns of simulation).

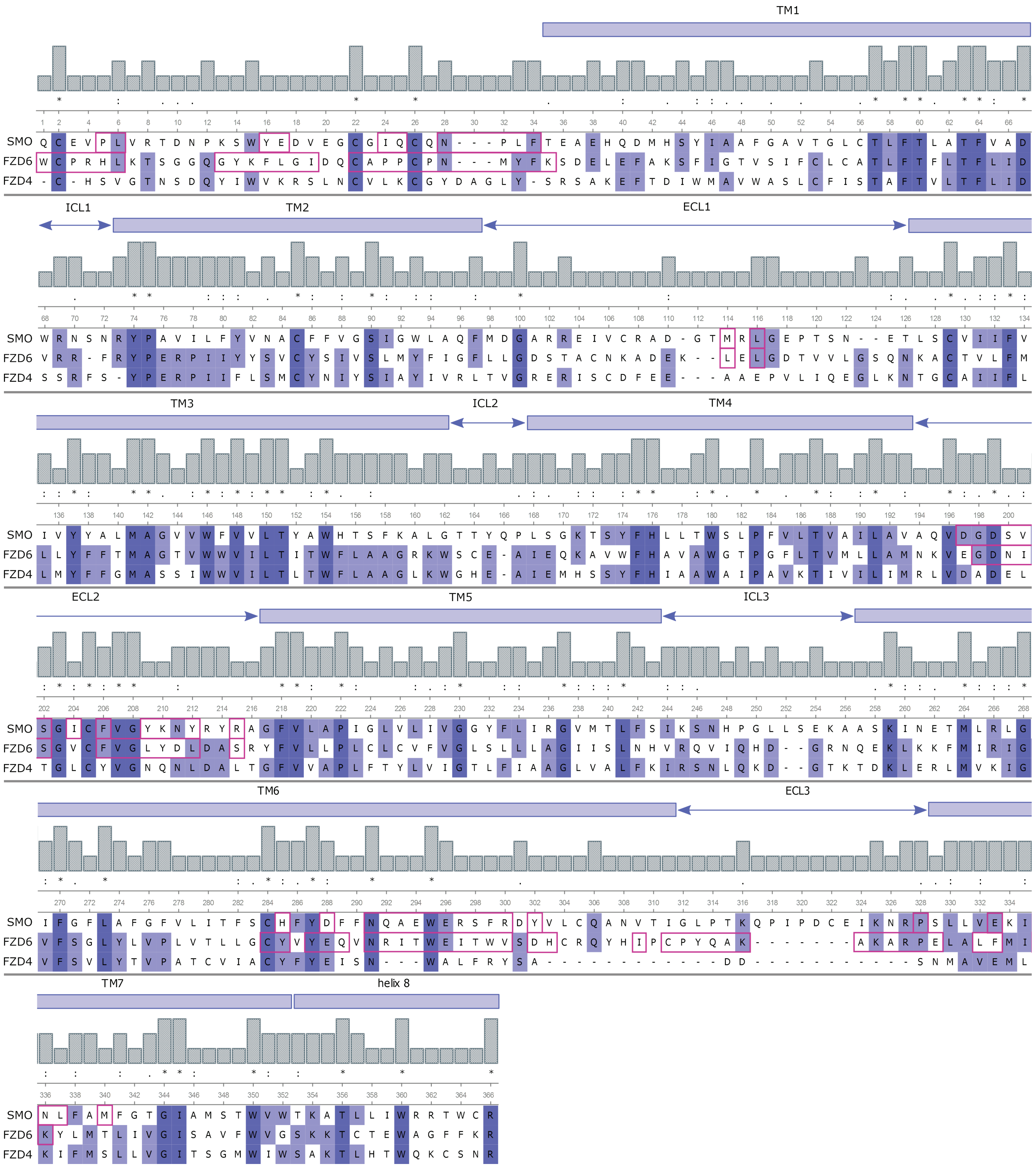

Supplementary Fig. 1

Supplementary Fig. 2. *In silico* analysis of SAG1.5-SMO and SAG1.3-SMO binding modes. a, Chemical structure of SAG1.5. **b**, RMSD plots for SAG1.3 and SAG1.5 in SMO. **c**, N219 distance plots for SAG1.3-SMO. **d**, D473$^{6.54}$ distance plots for SAG1.3-SMO. **e**, E518$^{7.38}$ distance plots for SAG1.3-SMO. The dotted line (red) indicates the maximum distance (4 Å) that is still likely to allow polar interactions. Thick traces indicate the moving average smoothed over a 2 ns window and thin traces represent raw data. RMSD values are calculated referred to the last frame of the 50 ns equilibration run (defined as t=0). The origin of the y axis for all graphs is 0 Å.

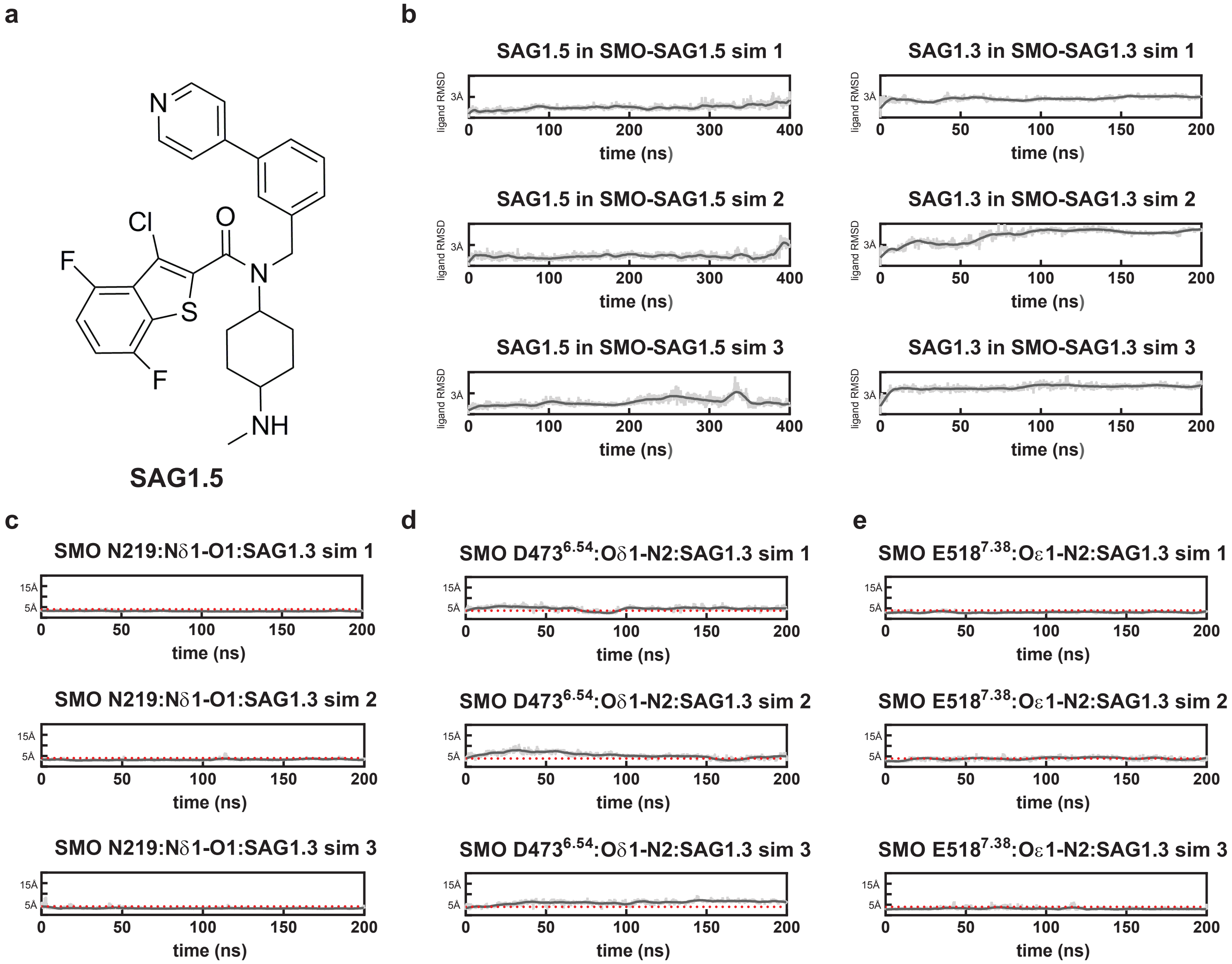

Supplementary Fig. 2

Supplementary Fig. 3. *In silico* analysis of SAG1.3-FZD$_6$ binding modes. a, E438$^{6.54}$ distance plots for SAG1.3-FZD$_6$ inactive model. **b**, N187 distance plots for SAG1.3-FZD$_6$ inactive model. **c**, R442$^{6.58}$ distance plots for SAG1.3-FZD$_6$ inactive model. **d**, E438$^{6.54}$ distance plots for SAG1.3-FZD$_6$ active-like model. **e.** D351 distance plots for SAG1.3-FZD$_6$ active-like model. **f**, RMSD plots for SAG1.3 in inactive FZD$_6$. **g**, RMSD plots of SAG1.3 in active-like FZD$_6$. **h**, N187 distance plots for SAG1.3-FZD$_6$ active-like model. The dotted line (red) indicates the maximum distance (4 Å) that is still likely to allow polar interactions. Thick traces indicate the moving average smoothed over a 2 ns window and thin traces represent raw data. RMSD values are calculated referred to the last frame of the 50 ns equilibration run (defined as t=0). The origin of the y axis for all graphs is 0 Å. **i**, SAG1.3 in active-like FZD$_6$ forms two water-mediated hydrogen bonds with N187 and D351 (both shown as sticks). One representative frame is shown with the red grids representing the location of water molecules found in over 80% of the MD frames of the active-like FZD$_6$ simulation 1 (1 µs). The hydrogen bonds are displayed as green dashed lines. The analysis was done in VMD using the VolMap tool. The displayed view is equal to that of Fig. 1d.

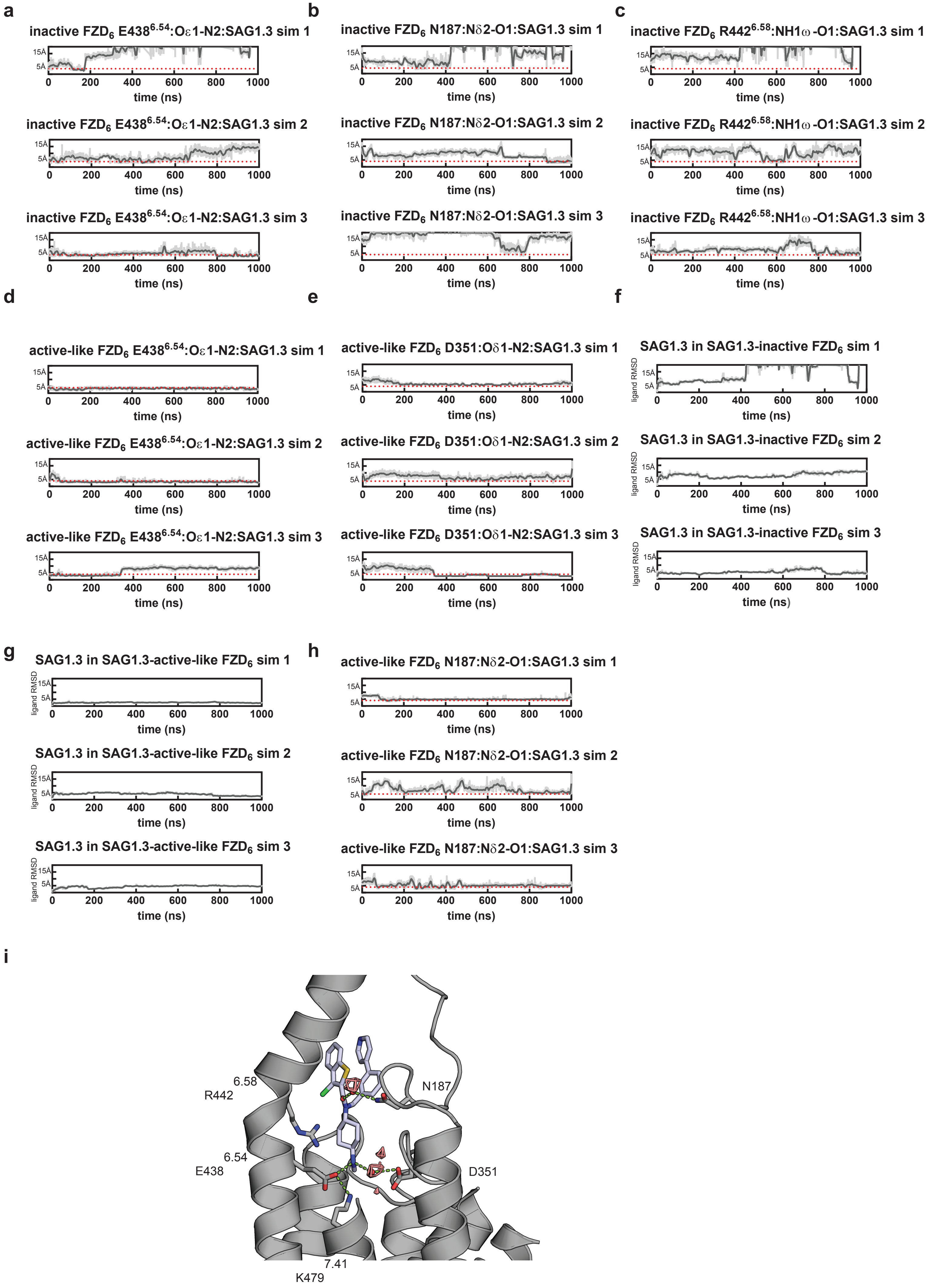

Supplementary Fig. 3

Supplementary Fig. 4. Dynamics of SAG1.3 interactions with SMO and FZD_6. Occurrence of interactions of SAG1.3/SAG1.5 with residues in SMO and FZD_6 is given as percentage of time when the distance between SAG1.3 and the depicted residues is <4 Å. The polar side chains involved in SAG-derivative interaction are highlighted in green/bold. Data were collected throughout the complete MD trajectories.

Residue in SMO	Residue in FZD_6	Location	SAG1.5-SMO	SAG1.3-SMO	SAG1.3-FZD_6 inactive model	SAG1.3-FZD_6 active-like model
GLY191	PHE159	N-term			15.65%	
GLU194	PRO162	N-term			14.66%	
PRO196	HIS164	N-term		13.98%		11.34%
LEU197	LEU165	N-term				16.31%
SER205	TYR173	N-term			61.95%	15.67%
TYR207	PHE175	N-term	45.34%	61.62%	15.70%	71.34%
GLU208	LEU176	N-term				59.55%
ILE215	PRO183	N-term	88.15%	49.16%	26.69%	57.70%
GLN216	PRO184	N-term			10.78%	
ASN219	ASN187	N-term	99.32%	99.23%		13.88%
PRO220	MET188	N-term	79.83%	63.92%		14.60%
LEU221	TYR189	N-term	81.17%	87.06%	94.18%	37.92%
PHE222	PHE190	N-term	18.42%	22.17%		
MET301	LEU267	ECL1	40.07%	28.05%		56.34%
LEU303	LEU269	ECL1	69.35%	38.23%		12.56%
ASP384	ASP351	ECL2	76.75%	54.89%		26.62%
SER385	ASN352	ECL2			12.19%	73.87%
VAL386	ILE353	ECL2			36.20%	40.02%
SER387	SER354	ECL2	30.09%	14.11%		
TYR394	LEU361	ECL2	98.92%	93.42%		61.69%
LYS395	TYR362	ECL2	81.50%	66.38%	46.72%	81.99%
ARG400	SER367	5.43	34.20%	33.60%		
HSD470	TYR435	6.51	19.93%		17.40%	
ASP473	GLU438	6.54	93.42%	40.51%	73.29%	75.64%
PHE474	GLN439	6.55			48.30%	
GLN477	ARG442	6.58	83.46%	24.87%	48.81%	83.23%
TRP480	TRP445	6.61	88.54%	57.29%	83.30%	80.06%
GLU481	GLU446	6.62	68.46%	34.32%	47.54%	75.40%
PHE484	TRP449	6.65	98.58%	94.90%	90.09%	93.72%
LEU498	TYR463	ECL3			67.59%	
PRO499	GLN464	ECL3			24.47%	
ARG512	ARG470	ECL3			14.03%	
PRO513	PRO471	ECL3	54.43%	66.25%	47.13%	78.87%
GLU518	PHE476	7.38	98.43%	91.47%		34.64%
ASN521	LYS479	7.41	18.33%			27.08%
LEU522	TYR480	7.42				20.38%

Supplementary Fig. 4

Supplementary Fig. 5. 2D plots of ligand interactions of SAG1.3 in SMO and FZD_6. Schematic 2D presentation of the ligand-receptor interactions visualized with the LigPlot$^+$ program[3]. **a**, SAG1.3 in complex with SMO. The polar interactions between N2 of SAG1.3 and $E518^{7.38}$ are highlighted. **b**, SAG1.3 in complex with inactive FZD_6. **c**, SAG1.3 in complex with active-like FZD_6. The polar interactions between N2 of SAG1.3 and $E438^{6.54}$ are highlighted. The LigPlots are based on the last frames of the MD presented in Fig. 1d.

a

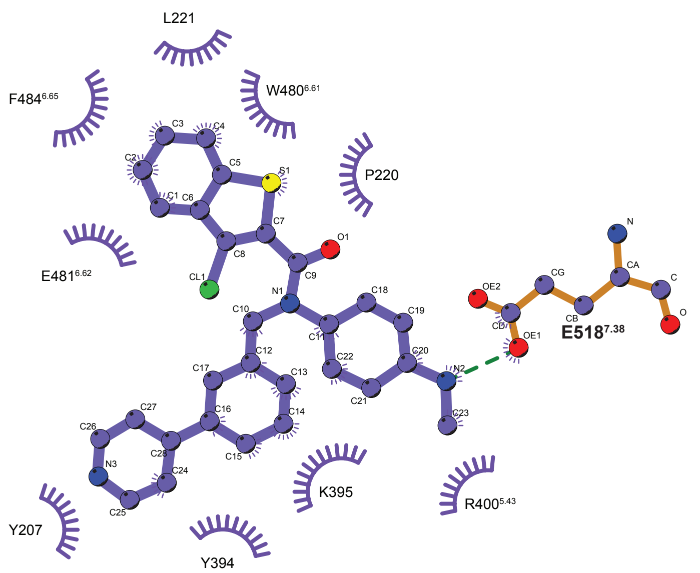

SAG1.3 bound to SMO

b

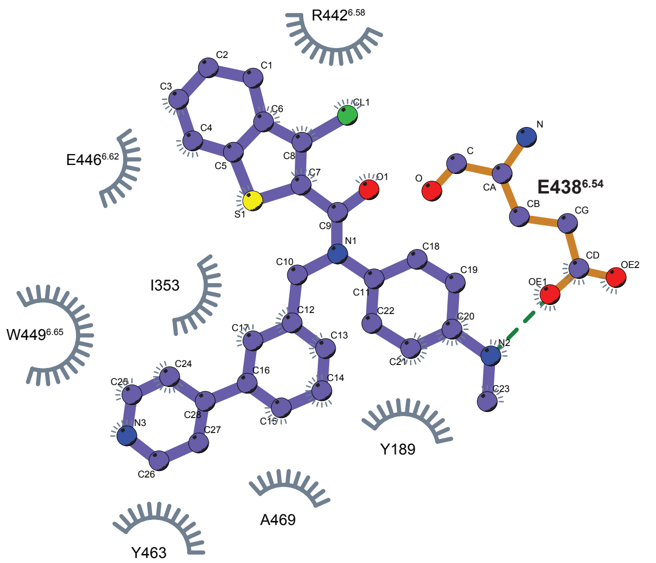

SAG1.3 bound to inactive FZD_6

c

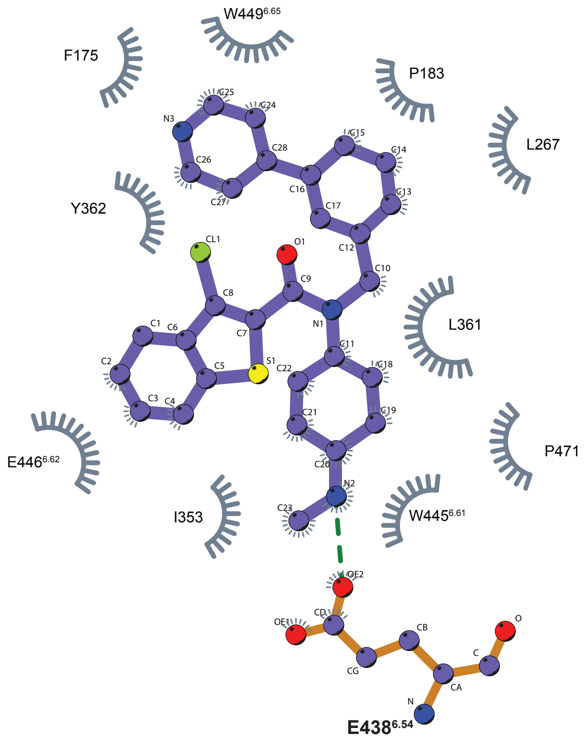

SAG1.3 bound to active-like FZD_6

Supplementary Fig. 5

Supplementary Fig 6. Protein Cα TM RMSD plots for all the MD simulations. RMSD plots (carbon Cα atoms of the transmembrane domains) for the independent simulations of ligand-free or ligand-bound human FZD_4 (**a**), FZD_6 (**b**, **c**, **d**, **e**) and SMO (**f**, **g**, **h**, **i**) as summarized in the Supplementary Fig. 7b. Thick traces indicate the moving average smoothed over a 2 ns window and thin traces represent raw data. RMSD values are calculated referred to the last frame of the 50 ns equilibration run (defined as t=0). The origin of the y axis for all graphs is 0 Å.

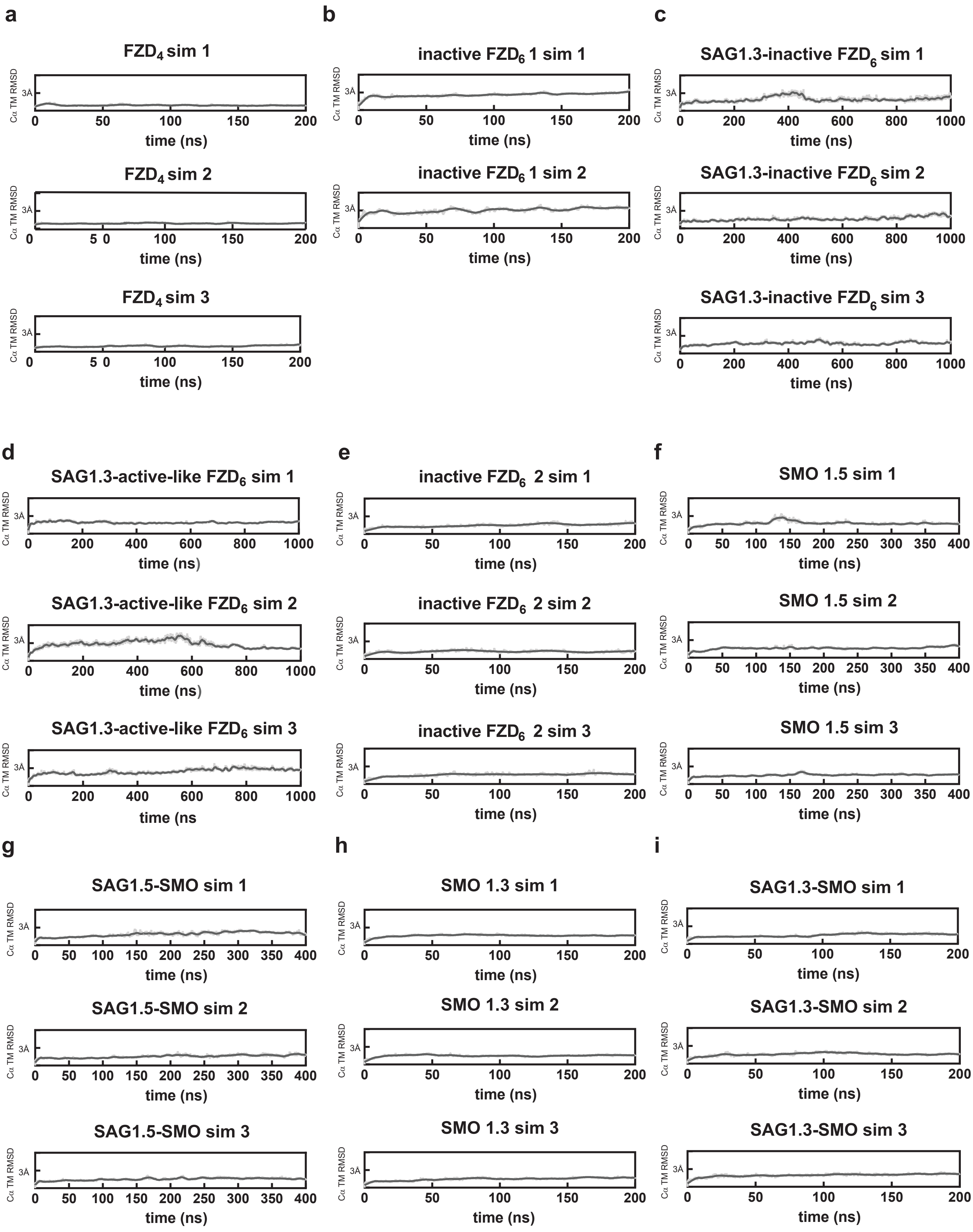

Supplementary Fig. 6

Supplementary Fig. 7. Supplementary information for the MD simulations. a, Representation of the membrane-inserted FZD_6 models. Protein is represented as violet cartoon and POPC lipids as sticks (carbon - white; oxygen - red; nitrogen - blue; phosphorus - orange). Water molecules, Na^+ and Cl^- are presented as red, yellow and green spheres, respectively. **b**, The table summarizes basic information on the MD simulations presented in this study.

a

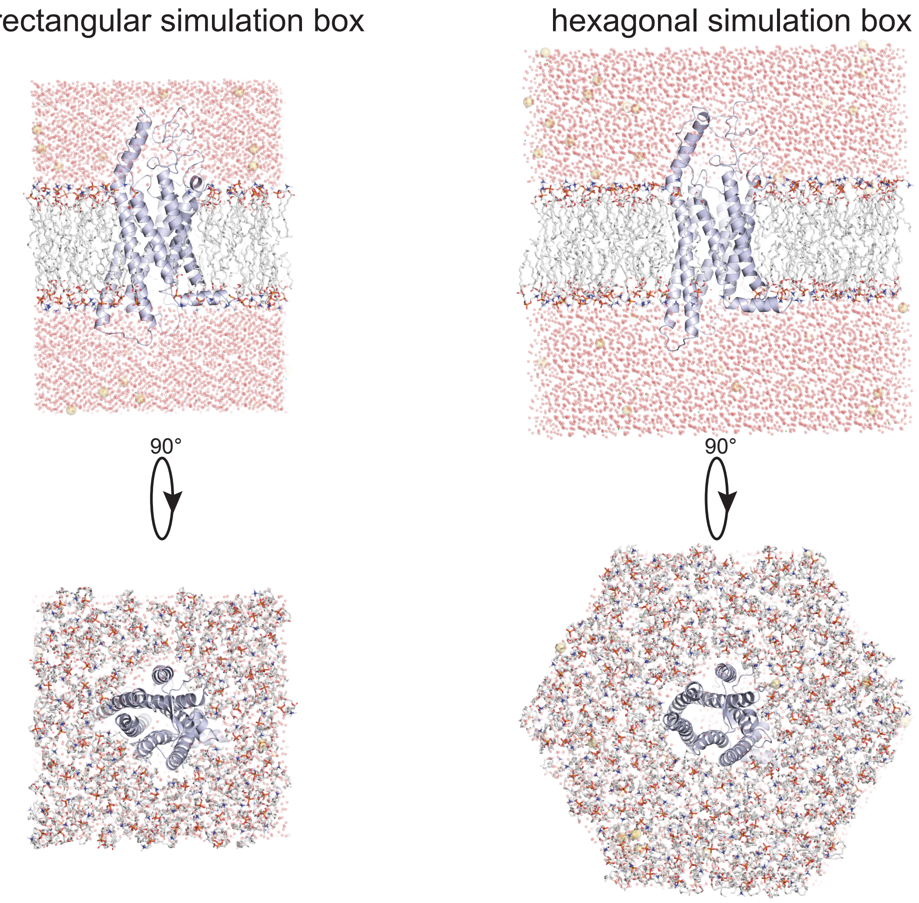

b

system simulated	initial conformation	duration of each production run	number of production runs	comments
apo-FZD_4	FZD_4 crystal structure, PDB ID: 6BD4	200 ns	3	
apo-FZD_6	homology model	200 ns	2	
SAG1.3-FZD_6	apo-FZD_6 after 200 ns, docked SAG1.3	1000 ns	3	
mock ligand-FZD_6	apo-FZD_6 after 200 ns, docked SAG1.3 substituted with mock ligand	200 ns	3	
apo-FZD_6	apo-FZD_6 after 200 ns	200 ns	3	
SAG1.3-active-like FZD_6	Homology model of FZD_6 (based on 6OT0), docked SAG1.3	1000 ns	3	Hexagonal simulation box, side 66 Å, height 129 Å (150 lipids / leaflet)
SAG1.3-active-like FZD_7	Homology model of FZD_7 (based on 6OT0), docked SAG1.3	500 ns	1	Hexagonal simulation box, side 66 Å, height 129 Å (150 lipids / leaflet)
apo-SMO	SMO crystal structure, PDB ID: 4QIN, SAG1.5 removed	400 ns	3	Simulation box size* 79 x 79 x 116 Å, no 50 ns equilibration run
SAG1.5-SMO	SMO crystal structure, PDB ID: 4QIN with SAG1.5	400 ns	3	Simulation box size* 79 x 79 x 116 Å, no 50 ns equilibration run
apo-SMO	SMO crystal structure, PDB ID: 4QIN, SAG1.5 removed	200 ns	3	
SAG1.3-SMO	SMO crystal structure, PDB ID: 4QIN, SAG1.5 substituted with SAG1.3	200 ns	3	

* standard simulation box size: 85 x 85 x 116 Å

Supplementary Fig. 7

Supplementary Fig. 8. Summary of all FZD constructs used in this study. Schematic summary of all human FZD_6 and FZD_4 constructs used in this study. Diverse tags: SNAP, FLAG, PK, FlAsH, His, Venus, TFP, *R*luc8, Nluc; ss at the N-terminus indicates presence of a signal sequence. CRD – cysteine rich domain**.**

Frizzled constructs

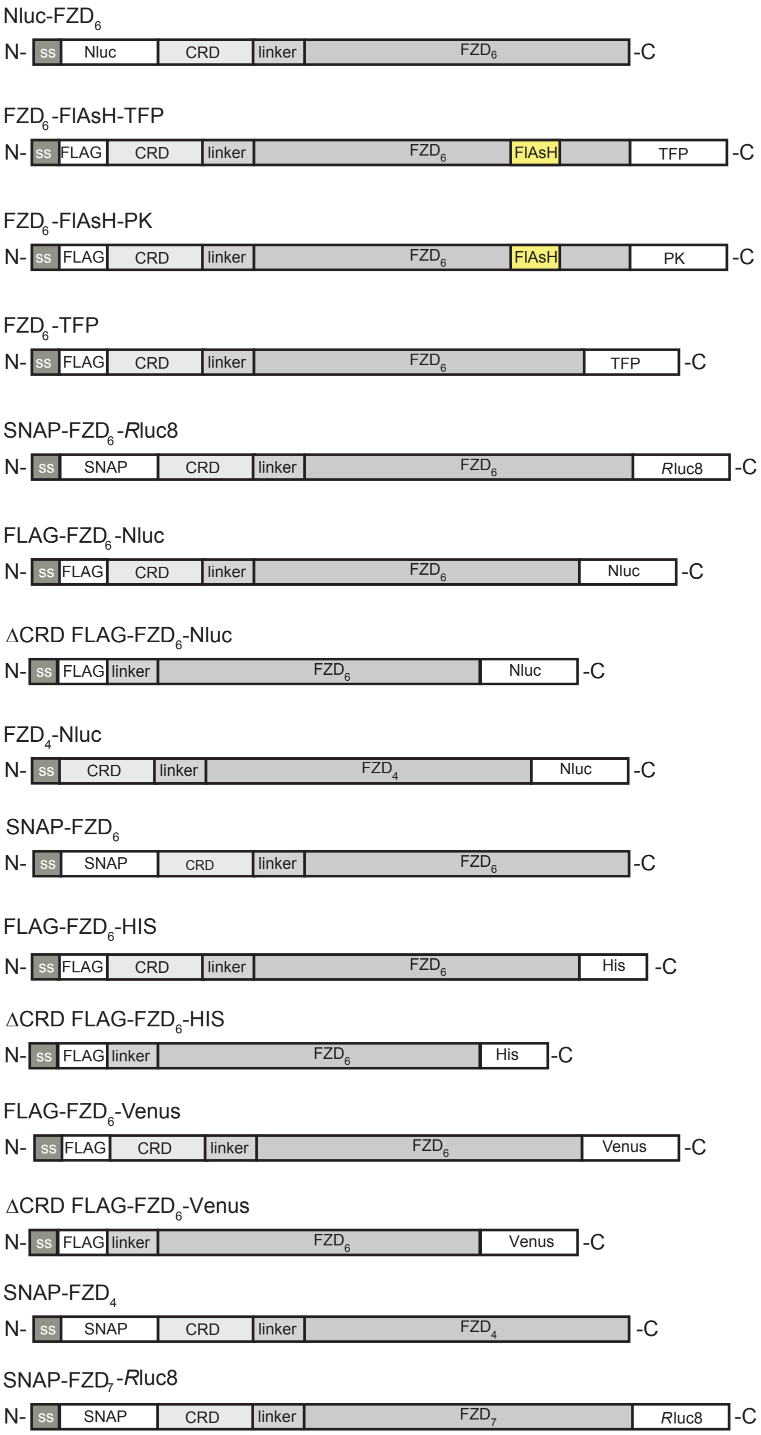

Supplementary Fig. 8

Supplementary Fig. 9. Generation of ∆SMO HEK293 cells with CRISPR-Cas9 genome editing. a, Target site for the single guide RNA (sgRNA) with the protospacer adjacent motif (PAM) highlighted in red in the human SMO gene. The translated human SMO protein sequence is shown below. **b**, The settings and gating of the fluorescence-activated cell sorting (FACS) of the transfected GFP^+ cells. **c**, Direct sequencing results. The first random frame-shift mutations occurring in the transfected cells are indicated with the blue arrows. **d**, The lack of SMO protein in the lysates of the generated ∆SMO HEK293 cells was validated by immunoblotting.

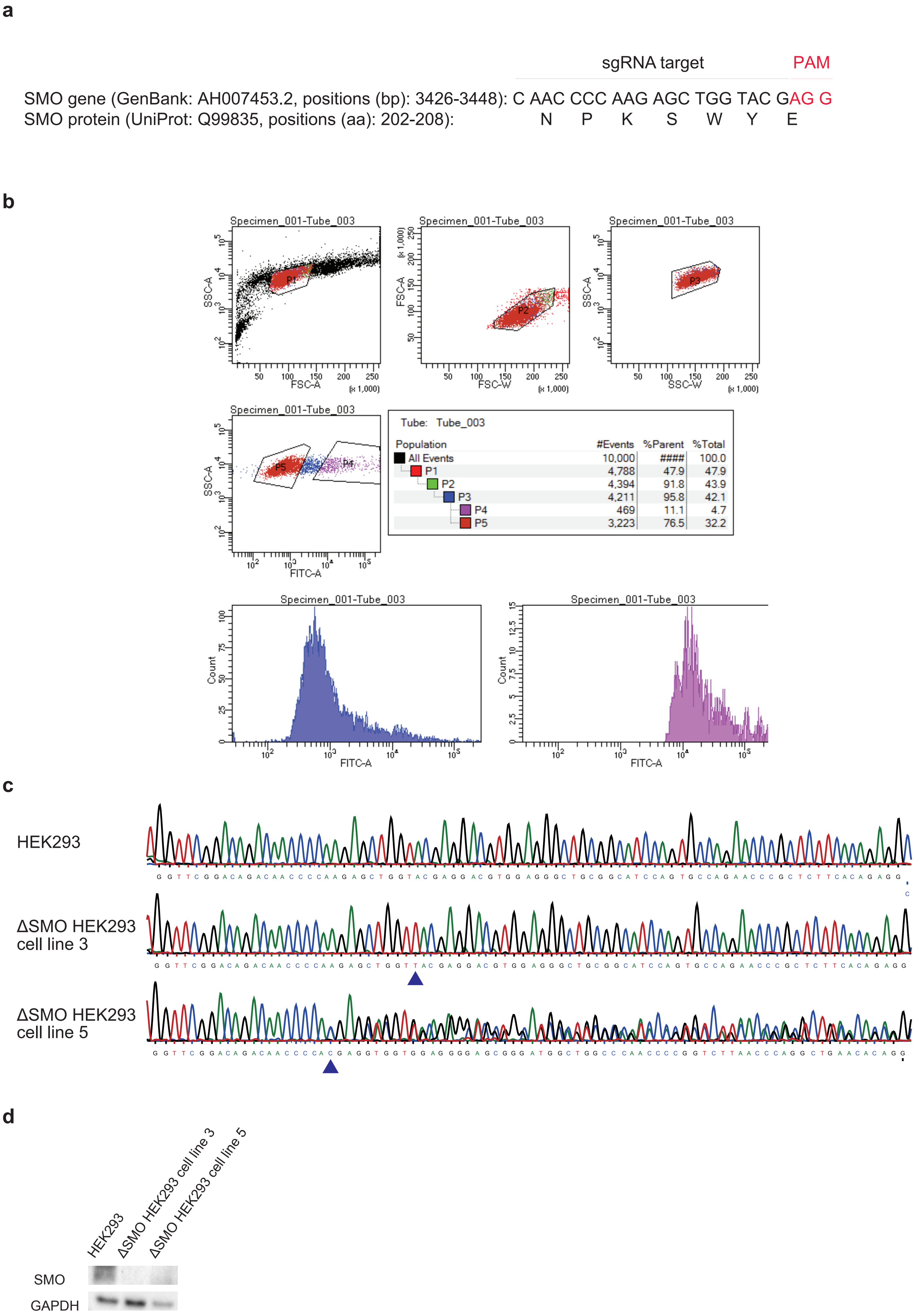

Supplementary Fig. 9

Supplementary Fig. 10. Validation of the BODIPY-cyclopamine/Nluc-FZD_6 NanoBRET binding assay. a, The Nluc-FZD_6 construct is efficiently trafficked to the cell surface. Nluc-A_3 was used as a positive control, and pcDNA and FLAG-FZD_6-Nluc were used as negative controls. The detection of the Nluc-tag was done with anti-Nluc antibody using live-cell ELISA. Data are presented as mean ± s.e.m. of n = 3-4 individual experiments; $F(3,11) = 11.17$. * $P < 0.05$, *** $P < 0.001$ represent comparisons with pcDNA (one-way ANOVA). **b**, molecular docking poses of cyclopamine in SMO and BODIPY-cyclopamine in FZD_6. The **top panel** represents cyclopamine-bound SMO (PDB ID: 4O9R, light blue sticks and cartoon) overlaid with SAG1.3-bound inactive FZD_6 (grey cartoon). The sphere represents the SAG1.3 location in the inactive FZD_6 (the top left view corresponds to the view in **Fig. 1d**). The **lower left panel** represents the BODIPY-cyclopamine-bound inactive FZD_6 with the ligand shown as gold sticks and the protein shown as grey surface. The **lower right panel** represents all the BODIPY-cyclopamine docking poses as generated by GLIDE with the highest scoring one highlighted. **c,** NanoBRET between Nluc-FZD_6 and BODIPY-cyclopamine is dependent upon BRET donor levels and acceptor:donor ratio and not linearly dependent on BRET acceptor levels. The Nluc-FZD_6 cell expression levels plot show the luminescence from donor-only wells as mean ± s.e.m. of n = 4 individual experiments; $P = 0.0058$; $t = 4.179$; $df = 6$, ** $P < 0.01$ (two-tailed unpaired *t*-test). The ΔBRET saturation curves show mean ± s.d. of each technical replicate of n=4 individual experiments. **d**, SAG1.3 (10 µM) displaces BODIPY-cyclopamine at Nluc-FZD_6. SAG1.3 pre-incubated for 30 minutes competes with the fluorescent ligand. Data are presented as mean ± s.e.m. of n = 3 individual experiments. **e,** Cyclopamine (10 µM) displaces BODIPY-cyclopamine at Nluc-FZD_6. Cyclopamine pre-incubated for 30 minutes competes with the fluorescent ligand. Data are presented as mean ± s.e.m. **f**, the **left** represents BODIPY-cyclopamine fluorescence values obtained prior to BRET reading for the experiments presented in Fig. 2c (left) and Supplementary Fig. 10d (right). The **middle** and **bottom** panels represent nanoluciferase counts from donor only wells for the experiments presented in Supplementary Fig. 10d.

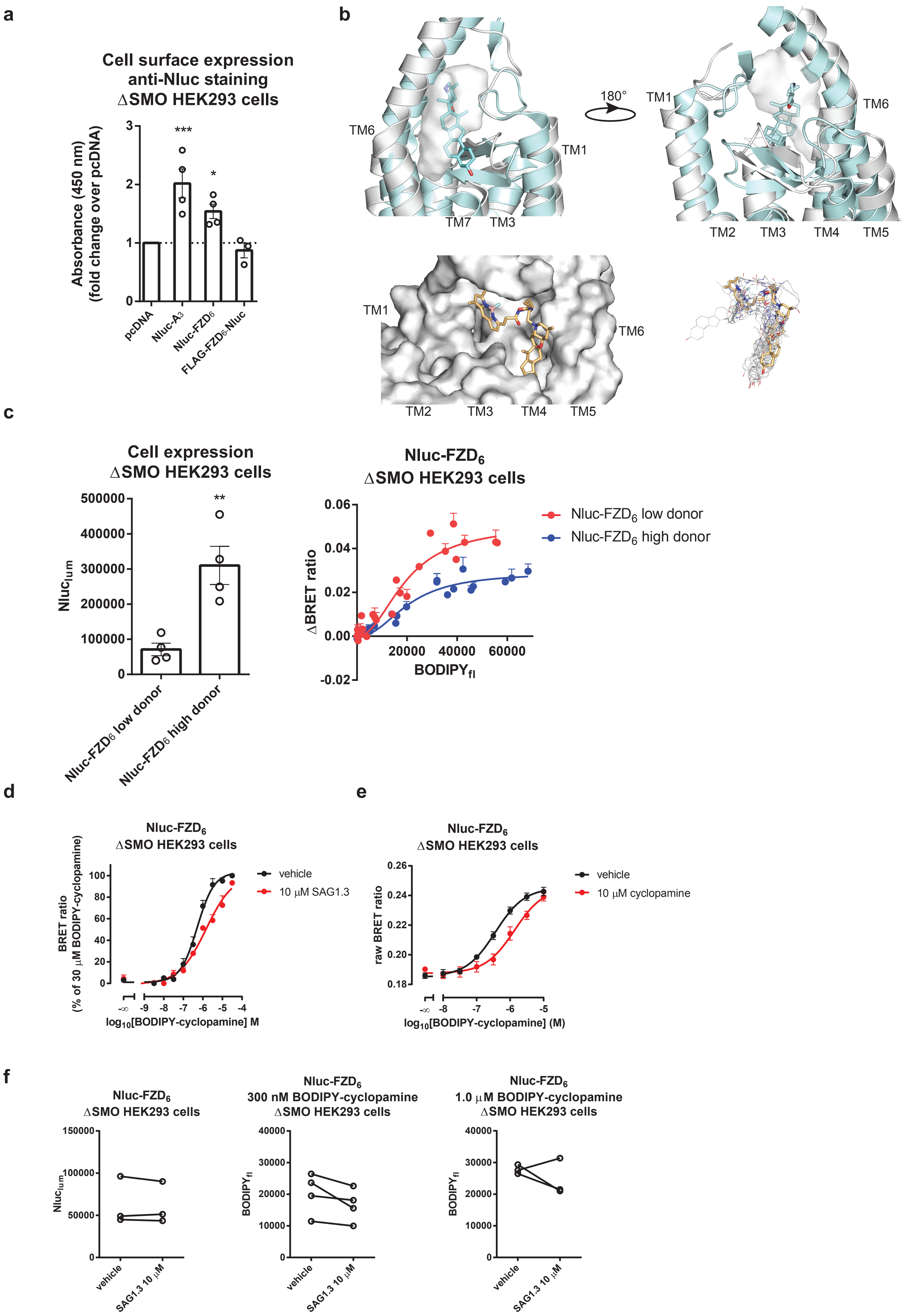

Supplementary Fig. 10

Supplementary Fig. 11. Validation of the FZD_6-FRET probe. a, Both FZD_6-TFP and FZD_6-FlAsH-TFP are properly processed and embedded in the plasma membrane. Micrographs present confocal images of HEK293 cells transiently transfected with FZD_6-TFP and FZD_6-FlAsH-TFP visualizing TFP fluorescence in living cells with prominent localization to the cell membrane. Size bar = 10 µm. **b**, FRET efficiency of the probe was determined by dequenching TFP fluorescence using BAL as antidote. The data present mean ± s.e.m. of n = 46 individual cells monitored on 8 independent experimental days. **c**, Individual traces of FlAsH fluorescence (top), TFP fluorescence (middle) and FRET ratio (FlAsH divided by TFP fluorescence) from the cells transfected with FZD_6-FlAsH-TFP or co-transfected with FZD_6-TFP and FZD_6-FlAsH. The data show representative traces from n = 12 individual cells (for each construct) monitored on 2 experimental days.

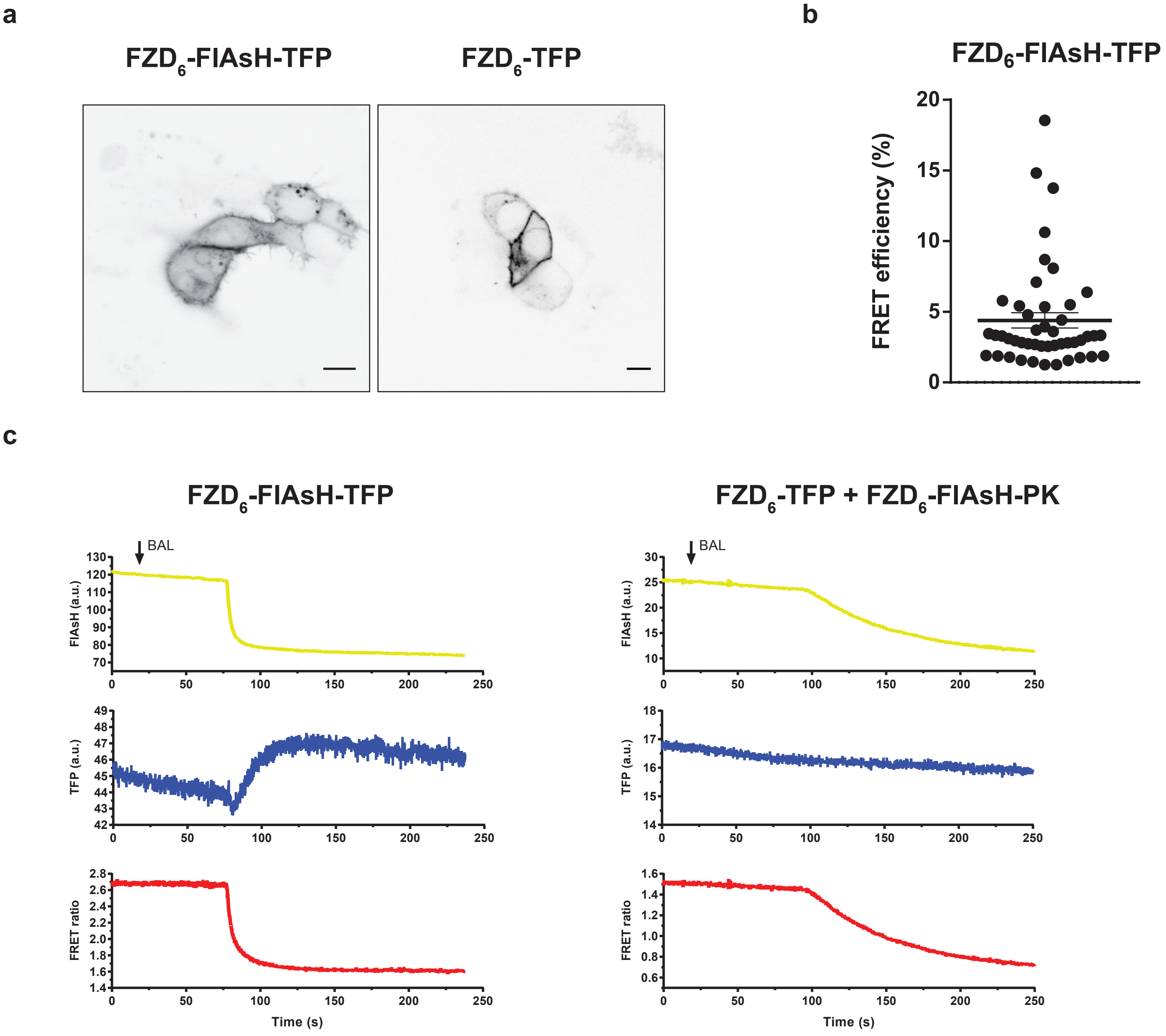

Supplementary Fig. 11

Supplementary Fig. 12. Cell surface expression of the FLAG-FZD$_6$ constructs. ∆CRD and full-length FLAG-FZD$_6$-Nluc, and ∆CRD and full-length FLAG-FZD$_6$-Venus are properly processed and embedded in the plasma membrane. FLAG-FZD$_6$-His constructs were used as positive controls[4] and pcDNA was used as a negative, empty vector control. The detection of the FLAG-tag was done with an anti-FLAG antibody using live-cell ELISA. Data are presented as mean ± s.e.m. of n = 3 individual experiments; $F(6,14) = 6.649$.* $P < 0.05$, ** $P < 0.01$, *** $P < 0.001$ represent comparisons with pcDNA (one-way ANOVA).

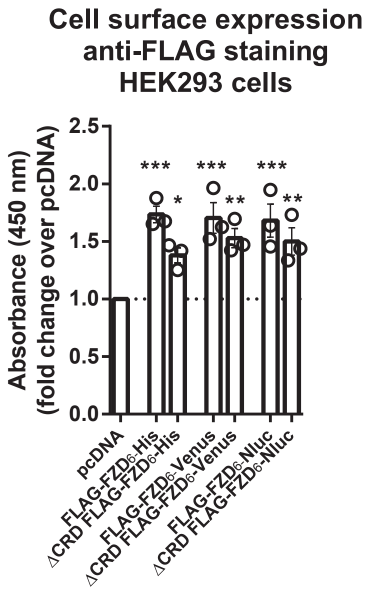

Supplementary Fig. 12

Supplementary Fig. 13. SAG1.3 functionally interacts also with FZD_7. a, Increasing concentrations of SAG1.3 induced a bell-shaped, concentration-dependent recruitment of the Venus-mGs to SNAP-FZD_7-*R*luc8 in accordance to the findings for FZD_6 and mGsi presented in Fig. 4d. **b**, 2D-sequence alignment of the SAG1.3 binding site of human FZD_6 and FZD_7 generated using ClustalX2 software[1] and visualized in UGENE[2]. Red squares indicate the amino acid residues (main chain or side chain) located within 4 Å from SAG1.3 in FZD_6 throughout the inactive and active-like MD simulations (observed after every 200th ns of simulation). **c**,3D-alignment of FZD_6 (white) and FZD_7 (salmon). The key residues of the binding site are depicted as sticks. - **d**, The last frame from the 500 ns of MD simulation of the SAG1.3-FZD_7 complex (**left panel**). Different positions of SAG1.3 throughout the time of simulation are indicated by transparent SAG1.3 molecules in the binding pocket. The view is corresponding to that of Fig 1d. RMSD plots for SAG1.3 in FZD_7 (**right panel**); $E492^{6.54}$ distance plots for SAG1.3-FZD_7; $R496^{6.58}$ distance plots for SAG1.3-FZD_7; N240 distance plots for SAG1.3-FZD_7; D405 distance plots for SAG1.3-FZD_7. The dotted line (red) indicates the maximum distance (4 Å) that is still likely to allow polar interactions. Thick traces indicate the moving average smoothed over a 2 ns window and thin traces represent raw data. RMSD values are calculated referred to the last frame of the 50 ns equilibration run (defined as t=0). The origin of the y axis for all graphs is 0 Å.

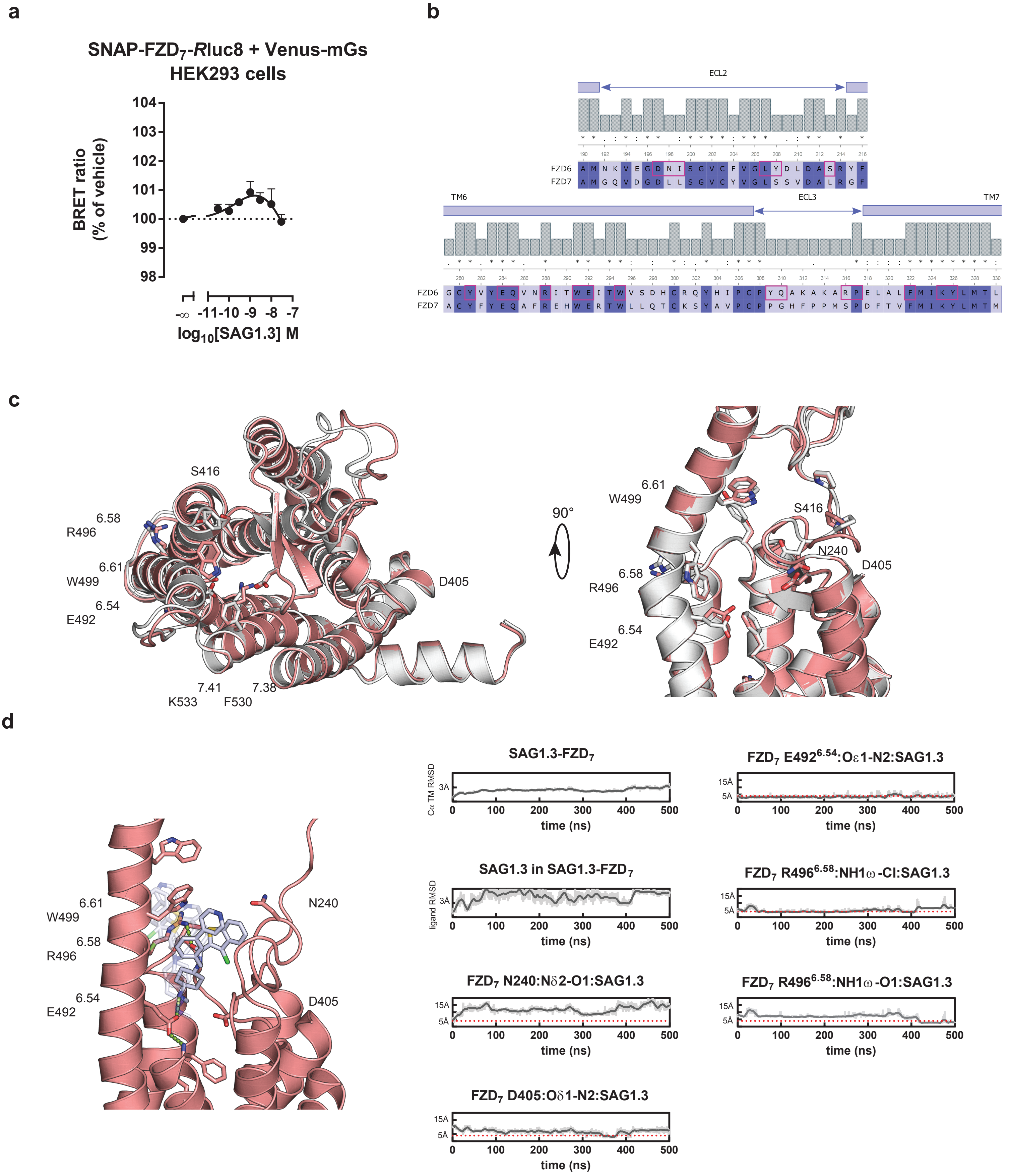

Supplementary Fig. 13

Supplementary Fig. 14. Validation of the NanoBiT G_i protein activation assay using the muscarinic M_2 receptor. a, ΔSMO HEK293 cells transiently transfected with the muscarinic M_2 receptor and the NanoBiT components for the heterotrimeric G_i protein ($G\alpha_{i1}$-LgBiT, SmBiT-$G\beta_5$, $G\gamma_2$) were stimulated with increasing concentrations of acetylcholine (ACh, 5 min) and the decrease in the Nluc luciferase signal was determined as described in the **Methods** section. Data are presented as mean ± s.e.m. of n = 4 individual experiments.

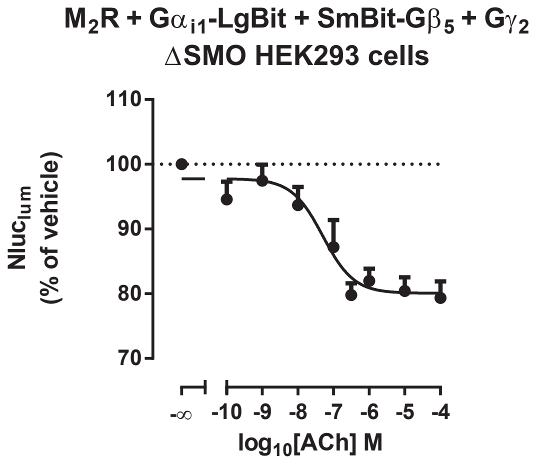

Supplementary Fig. 14

Supplementary Fig. 15. Validation of BRET-based DVL2 recruitment assay paradigms and SAG1.3 effects on FZD_6-DVL2 interactions. **a**, To quantify DVL2 membrane recruitment in ligand-free conditions, bystander BRET between Venus-KRas and Nluc-DVL2 was assessed over a range of acceptor:donor ratios in the presence of FLAG-FZD_6-His, ΔCRD FLAG-FZD_6-His or pcDNA as negative control (BRET saturation plot). The net BRET ratio values are presented as mean ± s.d. of n= 3 individual experiments. Overnight treatment with C59 (5 nM) had no significant effect neither on the bystander net BRET ratio nor on the co-transfected protein expression ratios (column plots). **b**, To quantify DVL2 membrane recruitment in ligand-free conditions, direct BRET between Nluc-DVL2 and FLAG-FZD_6-Venus, ΔCRD FLAG-FZD_6-Venus or Venus-N1 (negative control) was assessed over a range of acceptor:donor ratios (BRET saturation plot). The net BRET ratio values are presented as mean ± s.d. of n = 3 individual experiments. Overnight treatment with C59 (5 nM) had no significant effect neither on the direct net BRET ratio nor on the co-transfected protein expression ratio (column plots). **c**, Formation of phosphorylated and shifted DVL2 (PS-DVL2) in the SNAP-FZD_6-transiently transfected ΔSMO HEK293 cells was slightly enhanced by SAG1.3 (10 µM, 2 hours stimulation). The cells were serum-starved and treated with C59 (5 nM, overnight). Representative immunoblots are shown. Data are presented as mean ± s.e.m. of n = 3 individual experiments; $P = 0.0014$; $t = 7.890$; $df = 4$, ** $P < 0.01$ (two-tailed unpaired *t*-test).

a

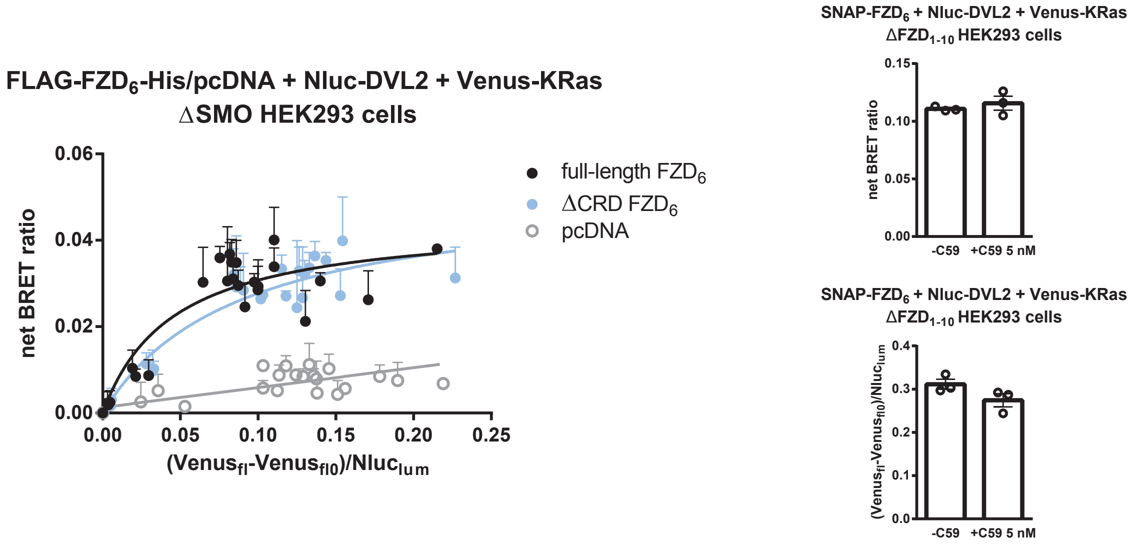

b

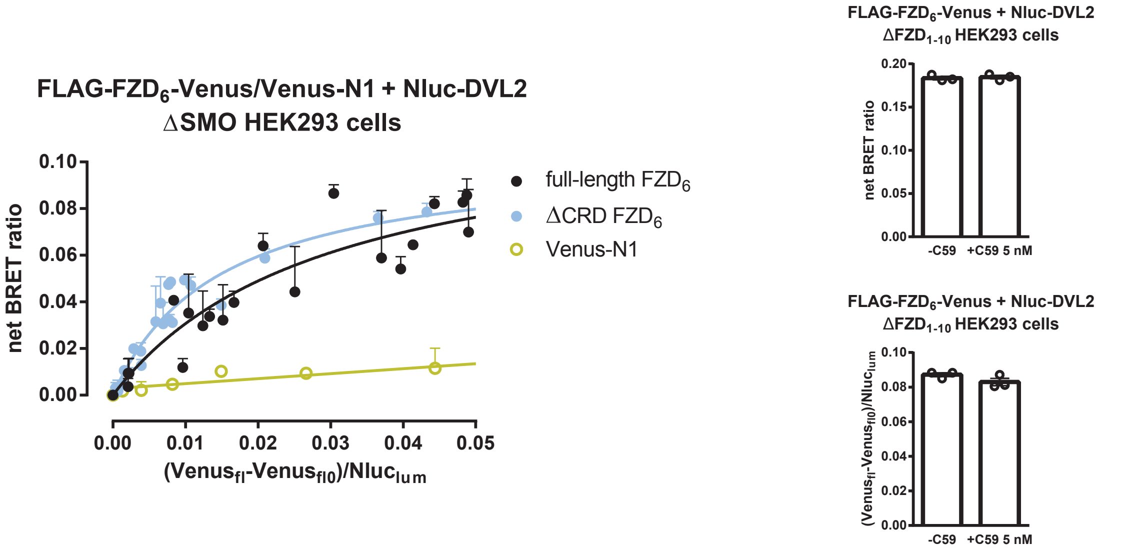

c

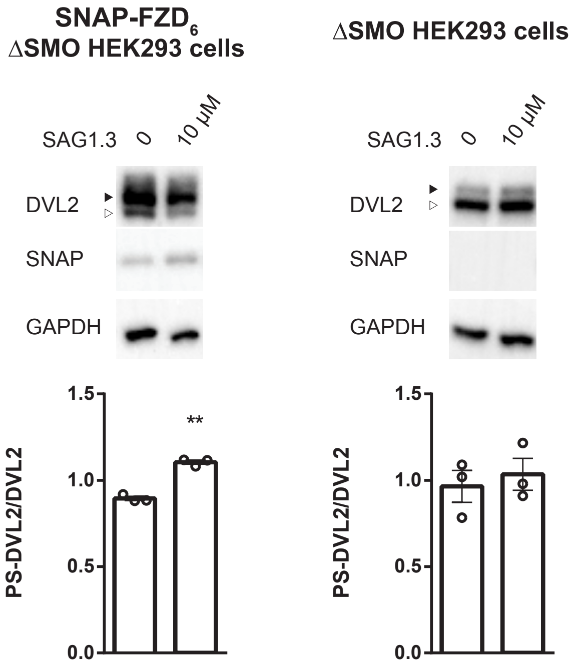

Supplementary Fig. 15

Supplementary Fig. 16. Site-directed mutagenesis supports the predicted location of SAG1.3 binding site on FZD_6. a, Mutations of polar residues potentially involved in SAG1.3 binding were generated by site-directed mutagenesis and tested for cell surface expression with SNAP-surface Alexa Fluor 647. Total cell expression of the SNAP-tagged proteins was assessed by immunoblotting using anti-SNAP and anti-GAPDH as loading control. Immunoblotting experiments were performed once for D351A, D351E, $R442A^{6.58}$ and $R442K^{6.58}$ mutants and twice for the other mutants and the wild type receptor (please also see Supplementary Fig 21). SNAP-surface Alexa Fluor 647 staining data are presented as mean ± s.e.m. of n = 3-5 individual experiments; $F(10,30) = 30.77$. ** (##) $P < 0.01$, **** (##) $P < 0.0001$. * denotes comparisons with pcDNA and # denotes comparisons with SNAP-FZD_6; (one-way ANOVA). **b**, Cell surface expression of the mutant receptor constructs mirrored their ability to recruit Nluc-DVL2 to the membrane as quantified by bystander BRET between Venus-KRas and Nluc-DVL2. Data are presented as mean ± s.e.m. of n = 3 individual experiments; $F(10,22) = 140.0$. **** (##) $P < 0.0001$. * denotes comparisons with pcDNA and # denotes comparisons with SNAP-FZD_6; (one-way ANOVA). Mutants that were expressed at the cell surface and were able to recruit Nluc-DVL2 to the membrane were assessed for their ability to respond to SAG1.3 in Venus-mGsi recruitment (**c**, n = 3-9 individual experiments), NanoBiT G_i protein dissociation (**d**, n = 4 individual experiments) and bystander BRET Nluc-DVL2 dynamics assays (**e**, n = 4 individual experiments).

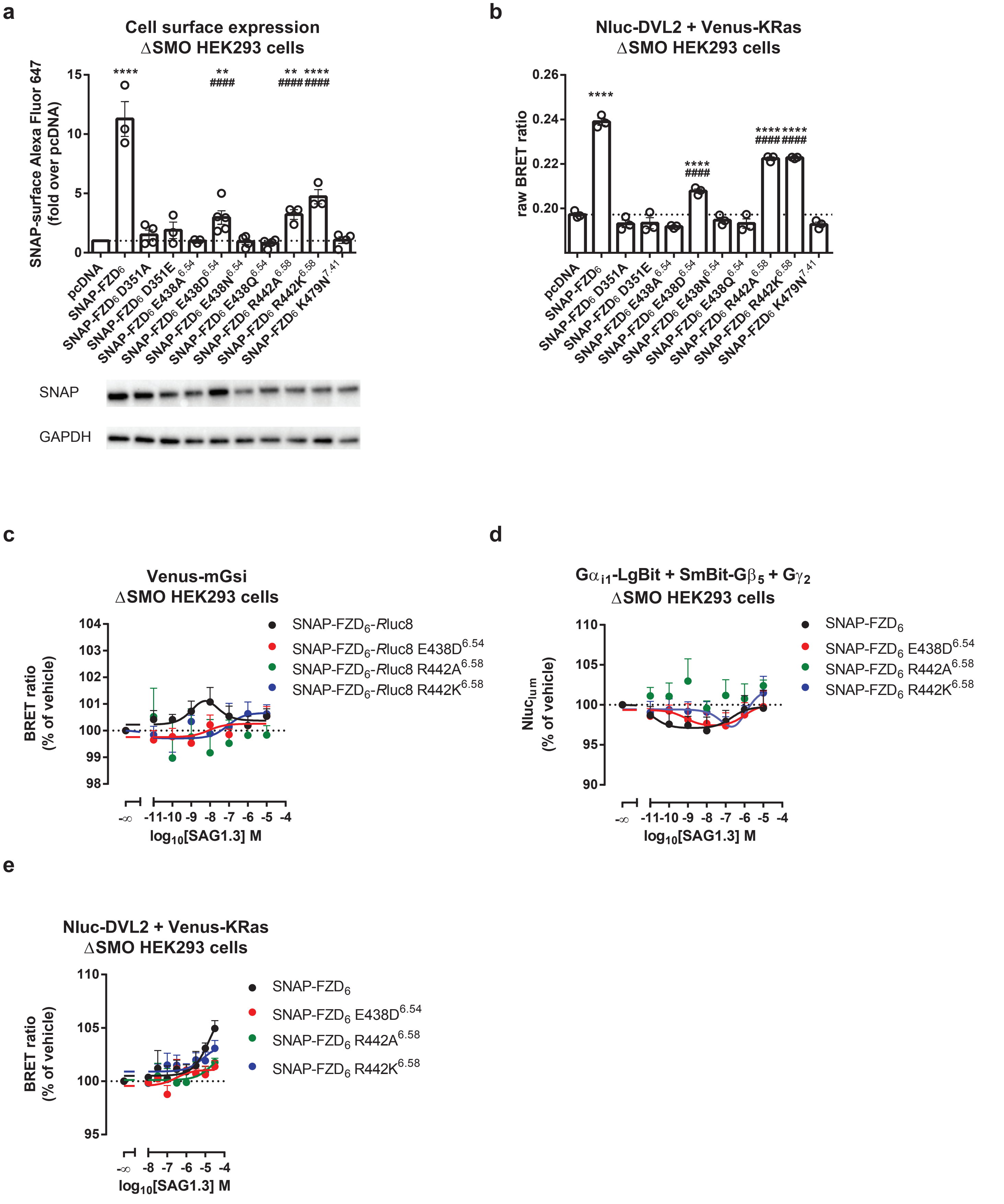

Supplementary Fig. 16

Supplementary Fig. 17. The summary of the mutagenesis approach to characterize the SAG1.3-FZD$_6$ binding site. The table summarizes the FZD$_6$ mutations in the SAG1.3 binding site that were validated for cell and cell surface expression, their ability to constitutively recruit Nluc-DVL2, interaction with mGsi in response to agonist and agonist-induced G$_i$ dissociation and Nluc-DVL2 dynamics. Only constructs expressed at the cell surface and able to recruit Nluc-DVL2 to the membrane were assessed in the mGsi, G$_i$ NanoBiT and bystander BRET Nluc-DVL2 recruitment assay. n.a. – not applicable; n.d. – not detectable; dash – not assessed. Percentages are estimates of responses in relationship to wild type FZD$_6$ (100%). The figures in the last two columns present the predicted hydrogen bond network in FZD$_6$ in the absence and presence of SAG1.3. The point mutations of FZD$_6$ were visualized in PyMOL. The ligand-free (apo) wild type FZD$_6$ is also presented in Supplementary Fig. 17. Data shown in Supplementary Fig. 15 provide the basis for the table.

FZD_6 mutant	Ballesteros-Weinstein coordinates	Cell expression (lysate)	Cell surface expression (SNAP)	DVL2 recruit ment	SAG1.3 in mGsi BRET	SAG1.3 in G_i NanoBiTs	SAG1.3 in DVL2 BRET	Hydrogen bond network in ligand-free FZD_6	SAG1.3 -FZD_6 complex – representative binding pose
wild type	n.a.	yes	100 %	100%	100%	100 %	100 %	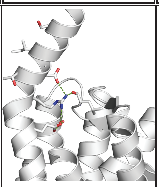	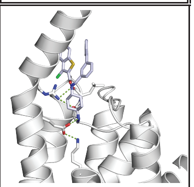
D351A	ECL2	yes	no	no	-	-	-		
D351E	ECL2	yes	no	no	-	-	-		
E438A	6.54	yes	no	no	-	-	-		
E438D	6.54	yes	~26%	~25%	~50% and shifted	~28% and shifted	~81% and shifted	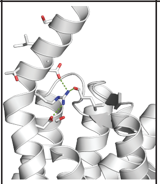	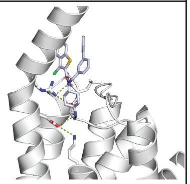
E438N	6.54	yes	no	no	-	-	-		
E438Q	6.54	yes	no	no	-	-	-		
R442A	6.58	yes	~28%	~60%	n.d.	~36% and shifted	n.d.	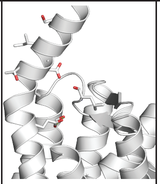	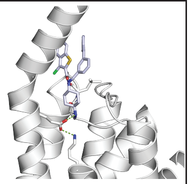
R442K	6.58	yes	~42%	~61%	~59% and shifted	~62% and shifted	~76% and shifted	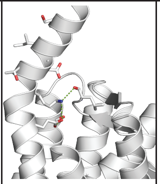	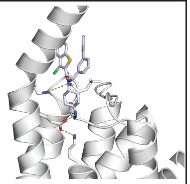
K479N	7.41	yes	no	no	-	-	-		

Supplementary Fig. 17

Supplementary Fig. 18. R442$^{6.58}$ is a key component in the SAG1.3 binding site of FZD$_6$. **a**, BODIPY-cyclopamine binding was quantified by NanoBRET detection in ΔSMO HEK293 cells transiently transfected (24 hr) with Nluc-FZD$_6$ (**a**) or Nluc-FZD$_6$ R442A$^{6.58}$ (**b**) in the absence or presence of 10 µM SAG1.3. Pre-incubation with SAG1.3 causes a significant (P = 0.0498) decrease in BODIPY-cyclopamine binding affinity to the wild-type receptor. Raw BRET ratio values for BODIPY-cyclopamine binding were normalized to 0 and 100 % to emphasize the curve shift. Binding data were fit to a three-parameter non-linear regression. Data originate from 4 independent experiments each performed at least in duplicates. Pre-normalization raw BRET ratio values (B_{max}) for BODIPY-cyclopamine are given in the table (**c**). The table summarizes the pK_d values for all conditions and pK_d values were compared using extra-sum-of-squares F test ($P<0.05$).

a

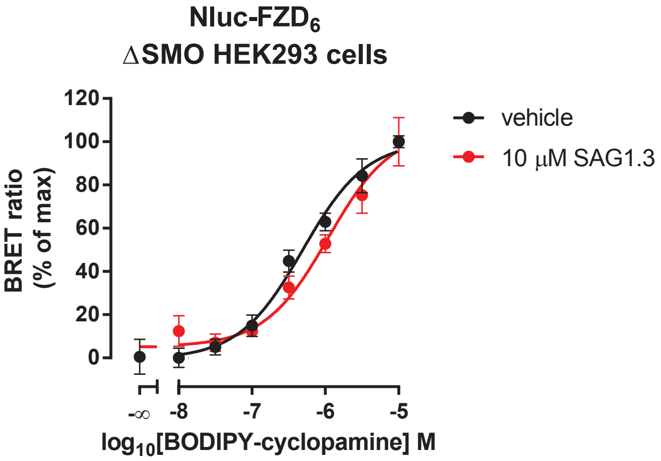

b

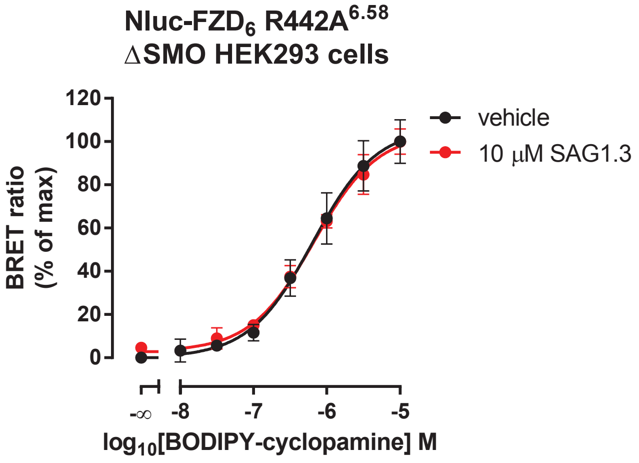

c

	pK_d ± s.d.		B_{max} ± s.d. (raw BRET ratio)	
	- SAG1.3	+SAG1.3	-SAG1.3	+SAG1.3
FZD_6	6.31 ± 0.10	5.96 ± 0.12	0.241 ± 0.003	0.241 ± 0.004
FZD_6 R442A$^{6.58}$	6.19 ± 0.14	6.18 ± 0.09	0.255 ± 0.005	0.243 ± 0.003

Supplementary Fig. 18

Supplementary Fig. 19. *In silico* analysis of the mock-ligand binding; Stabilization of the extramembraneous portion of the TM6. a, Chemical structure of the mock ligand, where the N2 of SAG1.3 was changed to a carbon. **b**, Plots comparing the relative distance over time in 3 independent MD simulations between the Oε1 of E438$^{6.54}$ of FZD_6 and the N2 in SAG1.3 or the C7 in the mock ligand. The dotted line (red) indicates the maximum distance (4 Å) that is still likely to allow polar interactions. Thick traces indicate the moving average smoothed over a 2 ns window and thin traces represent raw data. The origin of the y axis for all graphs is 0 Å. **c,** Comparison of the polar interactions stabilizing the extracellular portion of the TM6 in SMO (left) and inactive FZD_6 (right). Polar interactions between E438$^{6.54}$, S367$^{5.34}$, R442$^{6.58}$ and E446$^{6.62}$ interconnect the upper intramembrane part of TM6 with TM5 and the extramembraneous portion of TM6 in FZD_6. Additionally, in FZD_6 K479$^{7.41}$ is likely involved in the polar network as observed in the ligand-bound state (Supplementary Fig. 17). These interactions potentially contribute to the bend in TM6 and to the proper maturation and cellular trafficking of the receptor. The view and colour code correspond to the Fig. 1d.

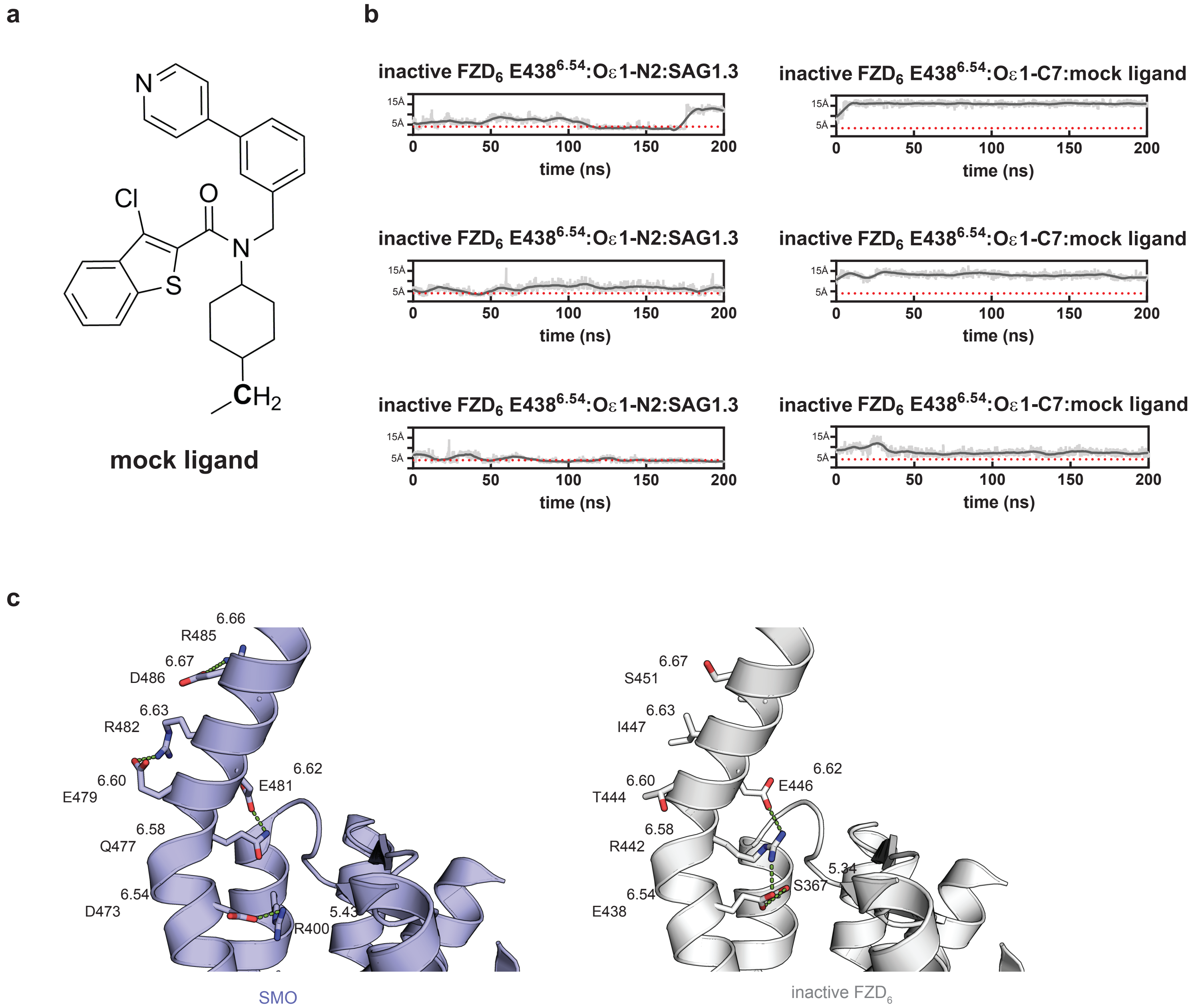

Supplementary Fig. 19

Supplementary Fig. 20. Purmorphamine as a FZD_6 agonist. a, Chemical structure of purmorphamine. **b**, Docking poses of purmorphamine in comparison to SAG1.3 in SMO (top) and inactive FZD_6 (bottom). The top-ranking poses of purmorphamine are shown as green sticks and the other docking poses as grey lines. The view and the colour code are corresponding to those of Fig. 1d. Docking poses are provided as mol2 files in Supplementary Data Files 32 and 33. Pharmacological profiling of purmorphamine action on SMO (**c**, n = 7 individual experiments) and FZD_6 (**d**, n = 3 individual experiments) in the BRET based Venus-mGsi recruitment assay. **e**, Increasing concentrations of purmorphamine did not affect FZD_6-DVL2 assembly in the SNAP-FZD_6/Nluc-DVL2 BRET assay (n = 3 individual experiments). Experiments shown in **d**, and **e** were performed in ΔSMO HEK293 cells. Data are presented as mean ± s.e.m.

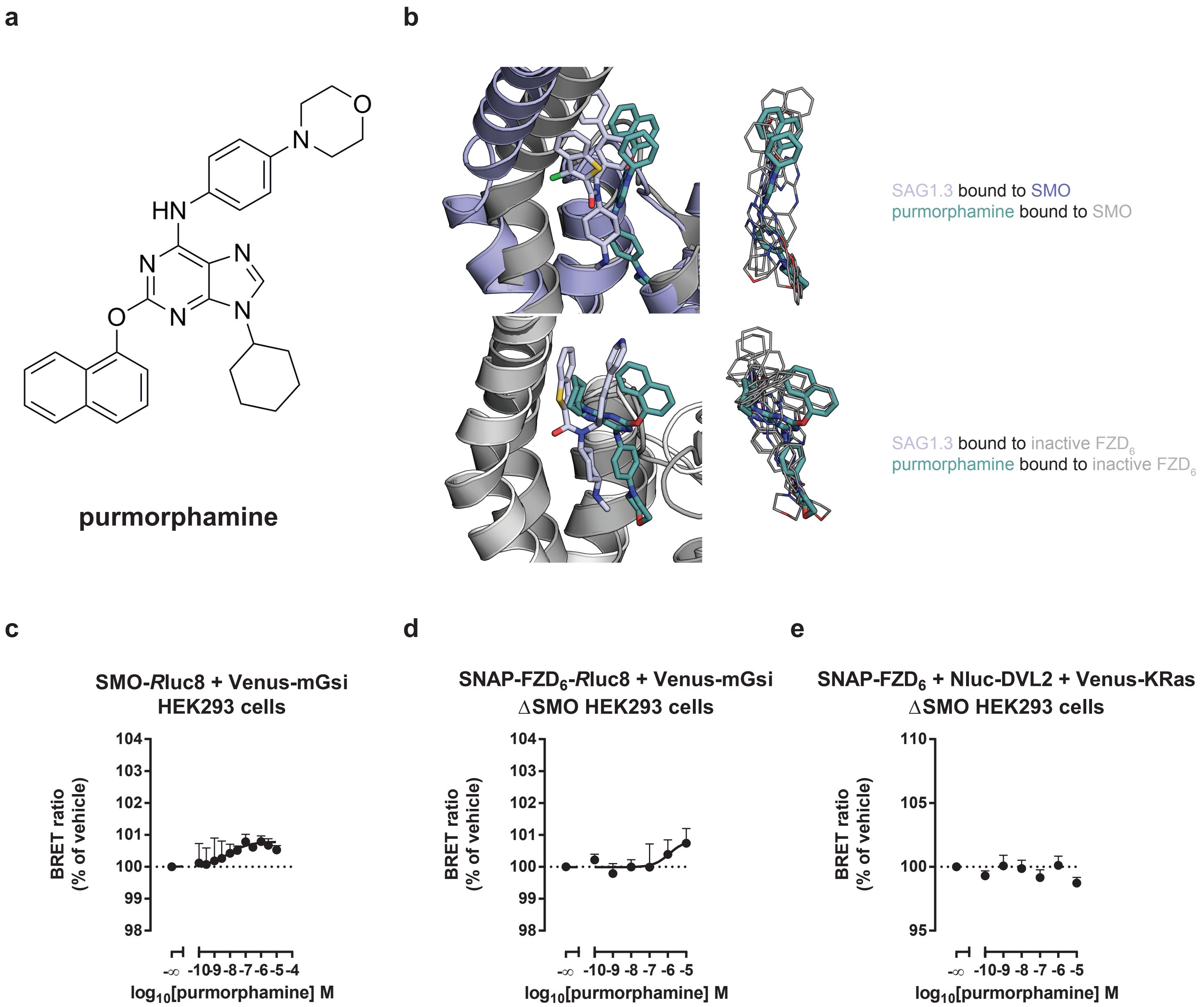

Supplementary Fig. 20

Supplementary Fig. 21. The statistical analysis of SAG1.3-induced responses in various assays used in this study. The assay window for the presented assays and the response induced by the partial agonist SAG1.3 is rather small. Here we present detailed data presentation and statistical analysis for all assays at selected SAG1.3 concentrations. The SAG1.3 concentrations inducing the highest response in each assay were selected. For the Nluc-DVL2 recruitment the lowest concentrations resulting in a statistically significant difference in the response comparing with the vehicle were selected. Two-tailed paired *t*-test (**b**) and two-tailed one-sample *t*-test (**a**, **c**-**m**) were used. For the data presented in **c** and **h**, which did not reach statistical significance in the two-tailed one-sample *t*-test, we also provide the P value as per Mann-Whitney test. Data are presented as mean values of individual experiments (**b**) or as mean values of individual experiments ± s.e.m (**a**, **c**-**m**); * P <0.05 and **** P < 0.0001.

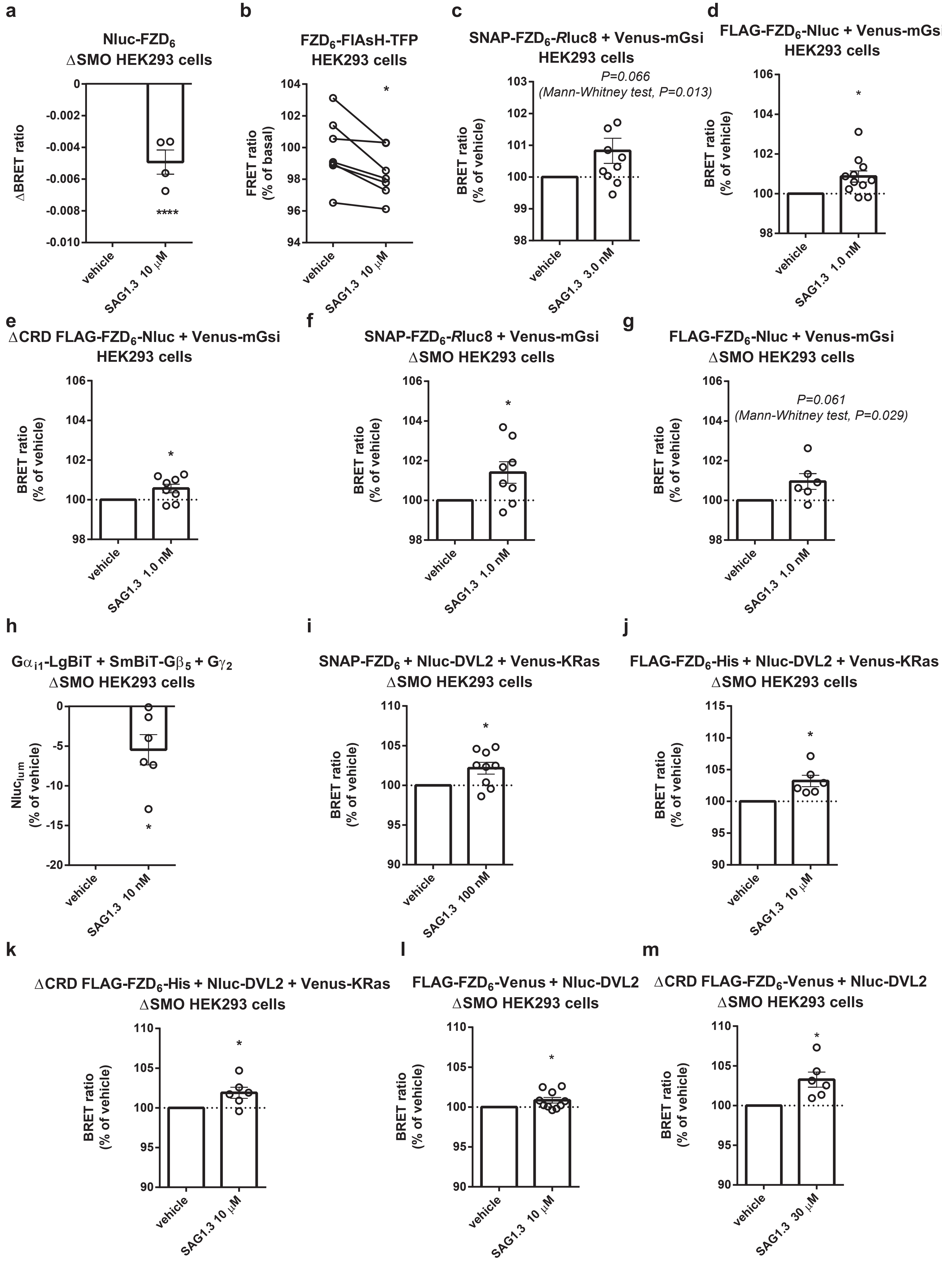

Supplementary Fig. 21

Supplementary Fig. 22. Pharmacological parameters of SAG1.3/SAG1.5-SMO, SAG1.3-FZD_6 and WNT-5A-FZD_6. a, Reported potencies and pharmacological profile of SAG derivatives on SMO-mediated signalling. **b**, Published binding affinities of SAG derivatives to SMO. **c**, SAG1.3 potencies (and binding affinity) on various assays in different cell systems reported in this study (mean ± s.d.). **d**, Potencies of recombinant, purified WNT-5A on various assays in different cell systems reported in this study (mean ± s.d.). dash – not assessed. References: [5-17]

a

assay	cell line	EC_{50}	concentration-response curve shape	reference
Gli reporter luciferase	C3H10T1/2	~ 30 nM for SAG1.3,<br>~ 3.0 nM for SAG1.5	bell-shaped (3.0 nM – 1.0 µM tested)	Frank-Kamenetsky et al., 2002
Gli reporter luciferase	Shh-LIGHT2	3.0 nM	bell-shaped (0.1 nM – 10 µM tested)	Chen et al., 2002
Inositol phosphate accumulation	HEK293	60 nM	bell-shaped (10 nM – 30 µM tested)	Masdeu et al., 2006
Alkaline phosphatase	C3H10T1/2	40 nM	-	Masdeu et al., 2006
Gli reporter luciferase	NIH-3T3	15 nM	-	Masdeau et al., 2006
Gli reporter luciferase	Shh-LIGHT2	50 nM	-	Masdeau et al., 2006
Gli reporter luciferase	Shh-LIGHT2	27 nM	fit to sigmoidal but appears bell-shaped (1.0 nM – 3.0 µM tested)	Wang et al., 2010
SMO/βarr2-GFP aggregate formation	U2OS	0.9 nM	sigmoidal (3.0 nM – 10 µM tested)	Wang et al., 2010
Gli reporter luciferase	Shh-LIGHT2	< 0.5 nM	bell-shaped (0.5 nM – 5.0 nM tested)	Bragina et al., 2010
Gli reporter luciferase	NIH 3T3	~ 1.0 nM	sigmoidal (0.1 nM – 10 nM tested)	Nachtergaele et al., 2012
Alkaline phosphatase	C3H10T1/2	130 nM	sigmoidal (10 nM – 3.0 µM tested)	Gorojankina et al., 2013
Gli reporter luciferase	Shh-LIGHT2	150 nM	sigmoidal (10 nM – 3.0 µM tested)	Gorojankina et al., 2013
Gli reporter luciferase	Shh-LIGHT2	~ 30 nM	sigmoidal (1.0 nM – 300 nM tested)	Nedelcu et al., 2013
Gli reporter luciferase	Shh-LIGHT2	~ 3.0 nM	bell-shaped (1.0 nM – 10 µM tested)	Khaliullina et al., 2015
Gli reporter luciferase	Shh-LIGHT2	~ 30 nM, SAG1.5 used	bell-shaped (0.1 nM – 10 µM tested)	Sever et al., 2016
mGsi recruitment	HEK293	0.2 nM and 2.1 nM	bell-shaped (0.1 nM – 100 nM tested)	Wright, Kozielewicz et al., 2019

b

assay	cell line	labelled ligand	K_i	reference
radioligand binding	HEK293	[^{3}H]-SAG1.5 at 0.4 nM	~ 10 nM for SAG1.3<br>~ 1.0 nM<br>for SAG1.5	Frank-Kamenetsky et al., 2002
radioligand binding	HEK293	[^{3}H]-SAG1.5 at 2.0 nM,	~ 1.0 nM for SAG1.3,<br>~ 0.5 nM<br>for SAG1.5	Rominger et al., 2009
radioligand binding	HEK293	[^{3}H]-cyclopamine at 7.0 nM	~ 3.7nM for SAG1.3,<br>~ 2.3 nM<br>for SAG1.5	Rominger et al., 2009
fluorescent binding	HEK293	Bodipy-cyclopamine at 25 nM	13 nM for SAG1.3,<br>15 nM for SAG1.5	Rominger et al., 2009
fluorescent binding	HEK293	Bodipy-cyclopamine at 5.0 nM	11 nM for SAG1.3	Wang et al., 2010
fluorescent binding	Cos-7	Bodipy-cyclopamine at 5.0 nM	~ 50 - 100 nM for SAG1.3	Wang et al., 2012
NanoBRET binding	ΔSMO HEK293	Bodipy-cyclopamine at 200 nM	955 nM for SAG1.3	Kozielewicz et al., 2019

c

assay	cell line	construct	SAG1.3 pEC_{50} (M)<br>(pK_i (M) for NanoBRET binding)
FRET binding	HEK293	FZD$_6$-FlAsH-TFP	6.5 ± 0.9
mGsi recruitment	HEK293	SNAP-FZD$_6$-*R*luc8	10.5 ± 1.0
mGsi recruitment	HEK293	FLAG-FZD$_6$-Nluc	10.5 ± 0.6
mGsi recruitment	HEK293	ΔCRD FLAG-FZD$_6$-Nluc	10.1 ± 1.0
mGsi recruitment	ΔSMO HEK293	SNAP-FZD$_6$-*R*luc8	10.7 ± 1.2
mGsi recruitment	ΔSMO HEK293	FLAG-FZD$_6$-Nluc	9.9 ± 1.2
mGsi recruitment	ΔSMO HEK293	ΔCRD FLAG-FZD$_6$-Nluc	9.3 ± 0.2
Gα_{11} NanoBiTs	ΔSMO HEK293	SNAP-FZD$_6$	9.8 ± 0.8
bystander BRET DVL2 recruitment	ΔSMO HEK293	SNAP-FZD$_6$	4.9 ± 0.3
bystander BRET DVL2 recruitment	ΔSMO HEK293	FLAG-FZD$_6$-His	5.0 ± 0.1
bystander BRET DVL2 recruitment	ΔSMO HEK293	ΔCRD FLAG-FZD$_6$-His	4.9 ± 0.1
direct BRET DVL2 recruitment	ΔSMO HEK293	FLAG-FZD$_6$-Venus	4.9 ± 0.2
direct BRET DVL2 recruitment	ΔSMO HEK293	ΔCRD FLAG-FZD$_6$-Venus	5.0 ± 0.2
NanoBRET binding	ΔSMO HEK293	Nluc-FZD$_6$	5.6 ± 0.1

d

assay	cell line	construct	WNT-5A $log_{10}EC_{50}$ (ng ml^{-1})
FRET binding	HEK293	FZD$_6$-FlAsH-TFP	2.2 ± 0.1
mGsi recruitment	HEK293	SNAP-FZD$_6$-*R*luc8	2.2 ± 0.3
mGsi recruitment	HEK293	FLAG-FZD$_6$-Nluc	2.6 ± 0.2
bystander BRET DVL2 recruitment	ΔFZD$_{1-10}$ HEK293	SNAP-FZD$_6$	0.2 ± 0.3
direct BRET DVL2 recruitment	ΔFZD$_{1-10}$ HEK293	FLAG-FZD$_6$-Venus	0.3 ± 0.3

Supplementary Fig. 22

Supplementary Fig. 23. Primer sequences. The sequences of all the primers used in the study.

primer name	forward/reverse	sequence
FZD_6_no_BamHI	forward	5’- AATTGACACTTTTGGAATCCGATGGCCTGAG-3’
FZD_6_no_BamHI	reverse	5’- CTCAGGCCATCGGATTCCAAAAGTGTCAATT-3
BamHI_no_ss_FZD_6	forward	5’- GACGGATCCCACAGTCTCTTCACCTGTGAA-3’
FZD_6_XbaI	reverse	5’-CGTTCTAGAAGTATCTGAATGACAACCACC-3’
BglII_no_ss_FZD_6	forward	5’-AAAAGATCTGCCACCATGAAGACG-3’
FZD_6_AgeI	reverse	5’- TTTACCGGTTGAGTATCTGAATGACAACC-3’
FZD_6_D351A	forward	5’-ACAAAGTTGAAGGAGCCAACATTAGTGGAGT-3’
FZD_6_D351A	reverse	5’-ACTCCACTAATGTTGGCTCCTTCAACTTTGT-3
FZD_6_D351E	forward	5’- CAAAGTTGAAGGAGAAAACATTAGTGGAGTT-3’
FZD_6_D351E	reverse	5’- AACTCCACTAATGTTTTCTCCTTCAACTTTG-3’
FZD_6_E438A	forward	5’-GATGTTACGTCTATGCGCAAGTGAACAGGAT-3’
FZD_6_E438A	reverse	5’-ATCCTG TTCACTTGCGCATAGACGTAACATC-3’
FZD_6_E438D	forward	5’- ATGTTACGTCTATGACCAAGTGAACAGGATT-3’
FZD_6_E438D	reverse	5’- AATCCTGTTCACTTGGTCATAGACGTAACAT-3’
FZD_6_E438N	forward	5’- GGATGTTACGTCTATAACCAAGTGAACAGGATT-3’
FZD_6_E438N	reverse	5’- AATCCTGTTCACTTGGTTATAGACGTAACATCC-3’
FZD_6_E438Q	forward	5’- GGATGTTACGTCTATCAGCAAGTGAACAGGA-3’
FZD_6_E438Q	reverse	5’- TCCTGTTCACTTGCTGATAGACGTAACATCC-3’
FZD_6_R442A	forward	5’- TATGAGCAAGTGAACGCGATTACCTGGGAGAT-3’
FZD_6_R442A	reverse	5’- ATCTCCCAGGTAATCGCGTTCACTTGCTCATA-3’
FZD_6_R442K	forward	5’- ATGAGCAAGTGAACAAGATTACCTGGGAGAT-3’
FZD_6_R442K	reverse	5’- ATCTCCCAGGTAATCTTGTTCACTTGCTCAT-3’
FZD_6_K479N	forward	5’- CTTTATTTATGATAAACTACCTGATGACATTA-3’
FZD_6_K479N	reverse	5’-TAATGTCATCAGGTAGTTTATCATAAATAAAG-3’
HindIII_FZD_6	forward	5’-GCCAAGCTTGCCACC ATGGAAATGTTTACATTTTTG-3’
FZD_6_ EcoRI	reverse	5’-CAGAATTCAGTATCTGAATGACAACC-3’
FZD_6_FlaSH_404_405	forward	5´- TGCCCGGGCTGCTGTATGGAGCCCCGGAACCAAGAAAAACTA- 3’
FZD_6_FlaSH_404_405	reverse	5´-AGCCCGGGCAACAGTTTAAAAAGCCATCATGTTGTATGAC-3’
BglII_FZD_6	forward	5’-AAAGATCTATGGAAATGTTTACATTTTTGTT-3’
FZD_6_ HindIII	reverse	5’-TTAAGCTTGCAGTATCTGAATGACAACCAC-3’

Supplementary Fig. 23

Supplementary Fig. 24. Uncropped immunoblots. Immunoblots for the data presented in Fig. 5, Supplementary Fig. 9, 15 and 16.

Uncroppped immunoblots for Fig. 5

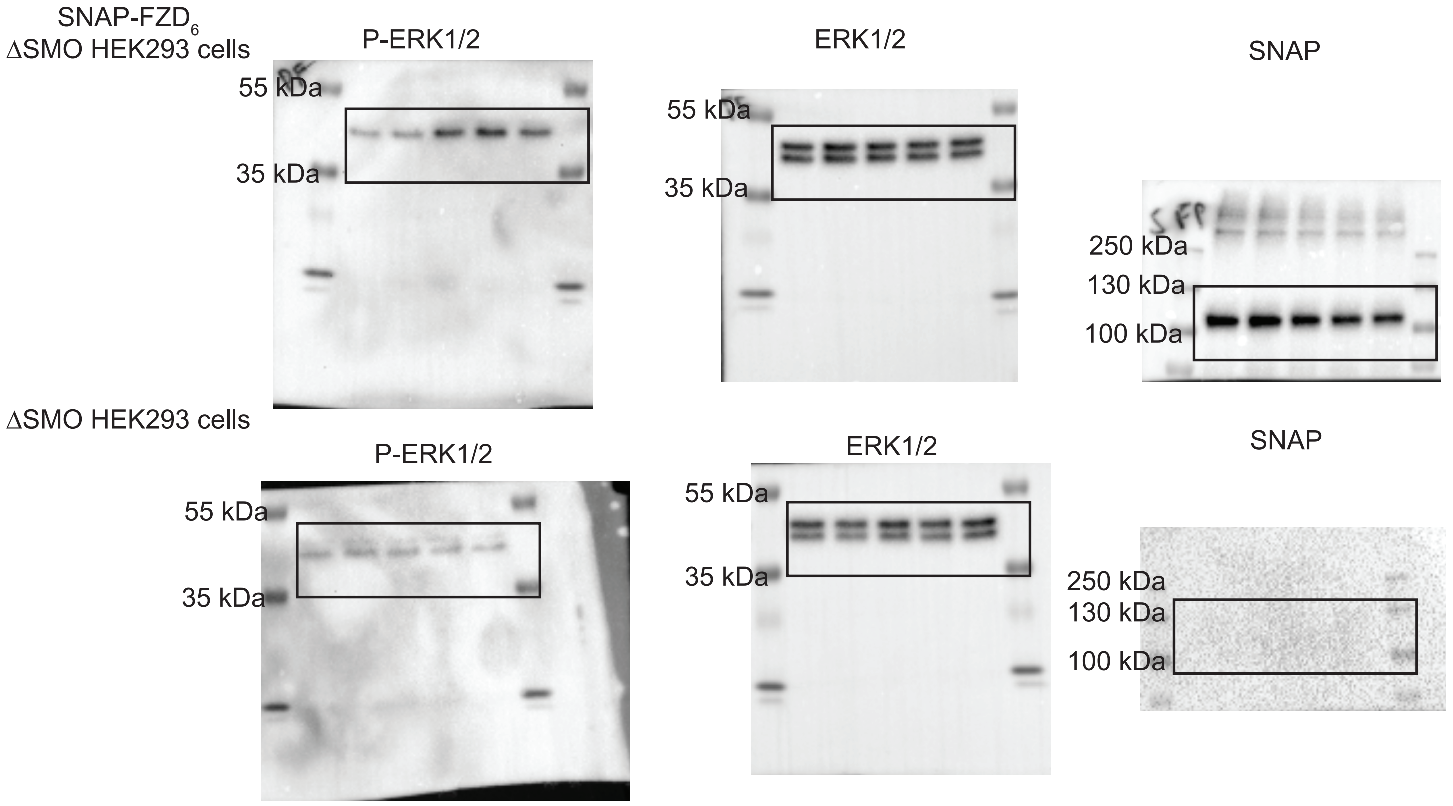

Uncroppped immunoblots for Supplementary Fig. 9

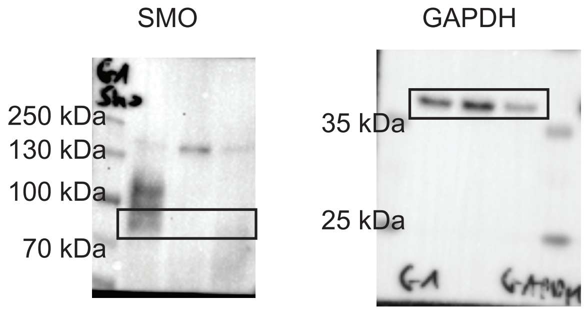

Uncroppped immunoblots for Supplementary Fig. 15

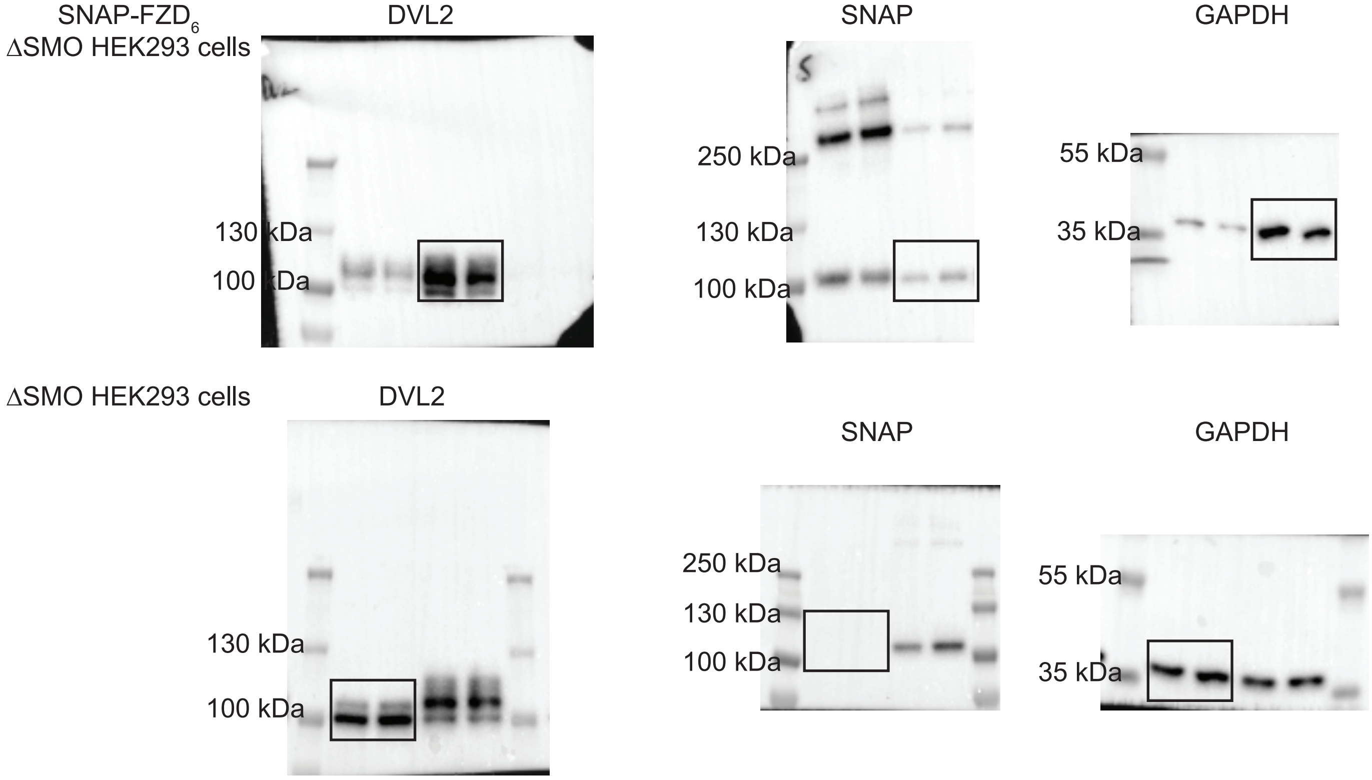

Supplementary Fig. 24 part 1

Uncroppped immunoblots for Supplementary Fig. 16

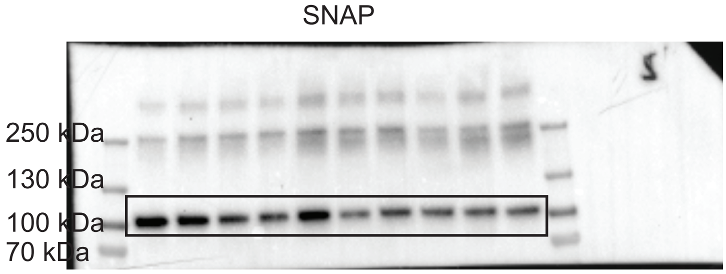

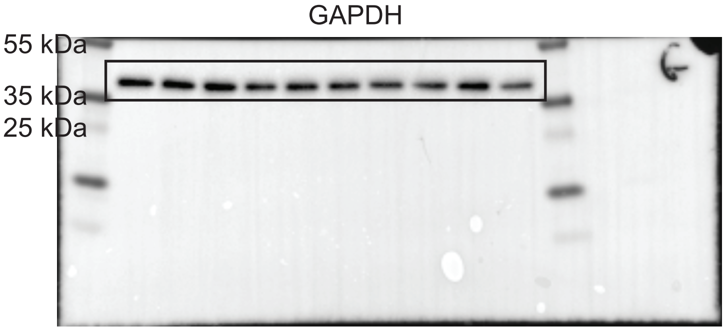

Additionally, we provide uncropped immunoblots for SNAP-FZD$_6$ wt, E438A$^{6.54}$, E438D$^{6.54}$, E438N$^{6.54}$, E438Q$^{6.54}$ and K479N$^{7.41}$ expression in ΔSMO HEK293 cells (together with pcDNA-transfected cells) as described in Supplementary Fig. 16.

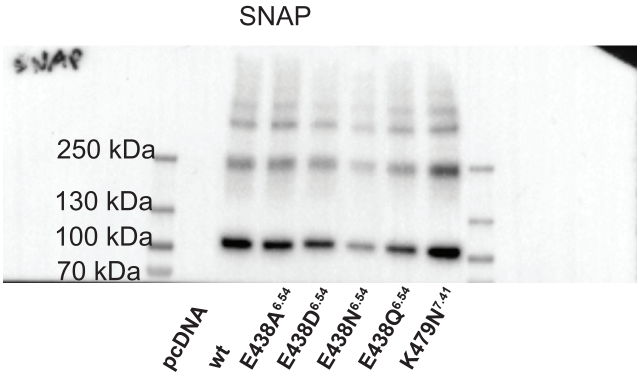

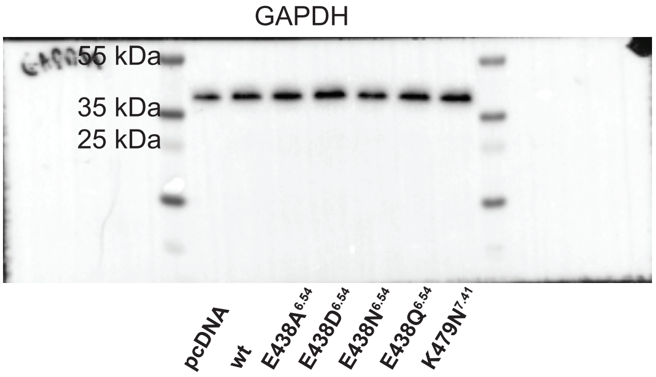

References

1. Larkin, M.A. et al. Clustal W and Clustal X version 2.0. *Bioinformatics* **23**, 2947-8 (2007).
2. Okonechnikov, K., Golosova, O., Fursov, M. & team, U. Unipro UGENE: a unified bioinformatics toolkit. *Bioinformatics* **28**, 1166-7 (2012).
3. Laskowski, R.A. & Swindells, M.B. LigPlot+: multiple ligand-protein interaction diagrams for drug discovery. *J Chem Inf Model* **51**, 2778-86 (2011).
4. Strakova, K. et al. Dishevelled enables casein kinase 1-mediated phosphorylation of Frizzled 6 required for cell membrane localization. J Biol Chem **293**, 18477-18493 (2018).
5. Frank-Kamenetsky, M. et al. Small-molecule modulators of Hedgehog signaling: identification and characterization of Smoothened agonists and antagonists. *J Biol* **1**, 10 (2002).
6. Chen, J.K., Taipale, J., Young, K.E., Maiti, T. & Beachy, P.A. Small molecule modulation of Smoothened activity. *Proc Natl Acad Sci U S A* **99**, 14071-6 (2002).
7. Masdeu, C. et al. Identification and characterization of Hedgehog modulator properties after functional coupling of Smoothened to G15. *Biochem Biophys Res Commun* **349**, 471-9 (2006).
8. Wang, J. et al. Identification of select glucocorticoids as Smoothened agonists: potential utility for regenerative medicine. *Proc Natl Acad Sci U S A* **107**, 9323-8 (2010).
9. Nachtergaele, S. et al. Oxysterols are allosteric activators of the oncoprotein Smoothened. *Nat Chem Biol* **8**, 211-20 (2012).
10. Gorojankina, T. et al. Discovery, molecular and pharmacological characterization of GSA-10, a novel small-molecule positive modulator of Smoothened. *Mol Pharmacol* **83**, 1020-9 (2013).
11. Nedelcu, D., Liu, J., Xu, Y., Jao, C. & Salic, A. Oxysterol binding to the extracellular domain of Smoothened in Hedgehog signaling. *Nat Chem Biol* **9**, 557-64 (2013).
12. Khaliullina, H., Bilgin, M., Sampaio, J.L., Shevchenko, A. & Eaton, S. Endocannabinoids are conserved inhibitors of the Hedgehog pathway. *Proc Natl Acad Sci U S A* **112**, 3415-20 (2015).
13. Sever, N. et al. Endogenous B-ring oxysterols inhibit the Hedgehog component Smoothened in a manner distinct from cyclopamine or side-chain oxysterols. *Proc Natl Acad Sci U S A* **113**(2016).
14. Wright, S.C. and Kozielewicz, P. et al. A conserved molecular switch in Class F receptors regulates receptor activation and pathway selection. *Nat Commun* **10**, 667 (2019).
15. Rominger, C.M. et al. Evidence for allosteric interactions of antagonist binding to the smoothened receptor. *J Pharmacol Exp Ther* **329**, 995-1005 (2009).
16. Wang, Y. et al. Glucocorticoid compounds modify smoothened localization and hedgehog pathway activity. *Chem Biol* **19**, 972-82 (2012).
17. Kozielewicz, P., Bowin, C.F., Turku, A. & Schulte, G. A NanoBRET-based binding assay for Smoothened allows real time analysis of ligand binding and distinction of two binding sites for BODIPY-cyclopamine. Mol Pharmacol **97**, 23-34 (2020).
