# Supplementary material for: Structural insight into small molecule action on Frizzleds
Source: Nat Commun. 2020 Jan 21;11:414. doi: 10.1038/s41467-019-14149-3 (PMC6972889; doi:10.1038/s41467-019-14149-3)
Supplement: Supplementary file 36 — Description of Additional Supplementary Files [file 41467_2019_14149_MOESM36_ESM.pdf]

Title: Supplementary Data 1
SMO in complex with SAG1.3 at a time point of 50 ns of simulation 1

Title: Supplementary Data 2
SMO in complex with SAG1.3 at a time point of 200 ns of simulation 1

Title: Supplementary Data 3
SMO in complex with SAG1.3 at a time point of 50 ns of simulation 2

Title: Supplementary Data 4
SMO in complex with SAG1.3 at a time point of 200 ns of simulation 2

Title: Supplementary Data 5
SMO in complex with SAG1.3 at a time point of 50 ns of simulation 3

Title: Supplementary Data 6
SMO in complex with SAG1.3 at a time point of 200 ns of simulation 3

Title: Supplementary Data 7
Inactive FZD6 model in complex with SAG1.3 at a time point of 50 ns of simulation

Title: Supplementary Data 8
Inactive FZD6 model in complex with SAG1.3 at a time point of 250 ns of simulation

Title: Supplementary Data 9
Inactive FZD6 model in complex with SAG1.3 at a time point of 450 ns of simulation

Title: Supplementary Data 10
Inactive FZD6 model in complex with SAG1.3 at a time point of 650 ns of simulation

Title: Supplementary Data 11
Inactive FZD6 model in complex with SAG1.3 at a time point of 850 ns of simulation

Title: Supplementary Data 12
Inactive FZD6 model in complex with SAG1.3 at a time point of 1000 ns of simulation

Title: Supplementary Data 13
Active-like FZD6 model in complex with SAG1.3 at a time point of 200 ns of simulation 1

Title: Supplementary Data 14
Active-like FZD6 model in complex with SAG1.3 at a time point of 400 ns of simulation 1

Title: Supplementary Data 15
Active-like FZD6 model in complex with SAG1.3 at a time point of 600 ns of simulation 1

Title: Supplementary Data 16
Active-like FZD6 model in complex with SAG1.3 at a time point of 800 ns of simulation 1

Title: Supplementary Data 17
Active-like FZD6 model in complex with SAG1.3 at a time point of 1000 ns of simulation 1 **Title: Title:**

Title: Supplementary Data 18
Active-like FZD6 model in complex with SAG1.3 at a time point of 200 ns of simulation 2

Title: Supplementary Data 19
Active-like FZD6 model in complex with SAG1.3 at a time point of 400 ns of simulation 2

Title: Supplementary Data 20
Active-like FZD6 model in complex with SAG1.3 at a time point of 600 ns of simulation 2

Title: Supplementary Data 21
Active-like FZD6 model in complex with SAG1.3 at a time point of 800 ns of simulation 2

Title: Supplementary Data 22
Active-like FZD6 model in complex with SAG1.3 at a time point of 1000 ns of simulation 2

Title: Supplementary Data 23
Active-like FZD6 model in complex with SAG1.3 at a time point of 200 ns of simulation 3

Title: Supplementary Data 24
Active-like FZD6 model in complex with SAG1.3 at a time point of 400 ns of simulation 3

Title: Supplementary Data 25
Active-like FZD6 model in complex with SAG1.3 at a time point of 600 ns of simulation 3

Title: Supplementary Data 26
Active-like FZD6 model in complex with SAG1.3 at a time point of 800 ns of simulation 3

Title: Supplementary Data 27
Active-like FZD6 model in complex with SAG1.3 at a time point of 1000 ns of simulation 3

Title: Supplementary Data 28
Active-like FZD7 model in complex with SAG1.3 at a time point of 100 ns of simulation

Title: Supplementary Data 29
Active-like FZD7 model in complex with SAG1.3 at a time point of 300 ns of simulation

Title: Supplementary Data 30
Active-like FZD7 model in complex with SAG1.3 at a time point of 500 ns of simulation

Title: Supplementary Data 31
Docking poses of Bodipy-cyclopamine to inactive FZD6 model

Title: Supplementary Data 32
mol2 Docking poses of purmorphamine to inactive FZD6 model

Title: Supplementary Data 33
Docking poses of purmorphamine to SMO

Title: Supplementary Data 34
6OT0 and FZD6 protein sequence alignment
