# Supplementary material for: Structural insight into small molecule action on Frizzleds
Source: Nat Commun. 2020 Jan 21;11:414. doi: 10.1038/s41467-019-14149-3 (PMC6972889; doi:10.1038/s41467-019-14149-3)
Supplement: Supplementary file 37 — Reporting Summary [file 41467_2019_14149_MOESM37_ESM.pdf]

nature research

Corresponding author(s): Gunnar Schulte
Last updated by author(s): Dec 4, 2019

Reporting Summary

Nature Research wishes to improve the reproducibility of the work that we publish. This form provides structure for consistency and transparency in reporting. For further information on Nature Research policies, see Authors & Referees and the Editorial Policy Checklist .

Statistics

For all statistical analyses, confirm that the following items are present in the figure legend, table legend, main text, or Methods section.

n/a	Confirmed	
☐	☒	The exact sample size (*n*) for each experimental group/condition, given as a discrete number and unit of measurement
☐	☒	A statement on whether measurements were taken from distinct samples or whether the same sample was measured repeatedly
☐	☒	The statistical test(s) used AND whether they are one- or two-sided<br>*Only common tests should be described solely by name; describe more complex techniques in the Methods section.*
☐	☒	A description of all covariates tested
☐	☒	A description of any assumptions or corrections, such as tests of normality and adjustment for multiple comparisons
☐	☒	A full description of the statistical parameters including central tendency (e.g. means) or other basic estimates (e.g. regression coefficient) AND variation (e.g. standard deviation) or associated estimates of uncertainty (e.g. confidence intervals)
☐	☒	For null hypothesis testing, the test statistic (e.g. *F*, *t*, *r*) with confidence intervals, effect sizes, degrees of freedom and *P* value noted<br>*Give P values as exact values whenever suitable.*
☒	☐	For Bayesian analysis, information on the choice of priors and Markov chain Monte Carlo settings
☒	☐	For hierarchical and complex designs, identification of the appropriate level for tests and full reporting of outcomes
☒	☐	Estimates of effect sizes (e.g. Cohen's *d*, Pearson's *r*), indicating how they were calculated

Our web collection on statistics for biologists contains articles on many of the points above.

Software and code

Policy information about availability of computer code

Data collection	Reader Control Software and Mars for BMG CLARIOstar plate reader, Gen5 for Synergy 2 plate reader, BD FACSDiva software for BD FACSDiva flow cytometer, ZEISS ZEN and Clampex for ZEISS Axiovert200 microscope, Image Lab for BioRad ChemiDoc, AutoDock Vina and GROMACS for molecular docking and molecular dynamics
Data analysis	GraphPad Prism 6, VMD 1.9.x, PyMol 2.0, MS Excel 2007 or 2013, Image Lab.

For manuscripts utilizing custom algorithms or software that are central to the research but not yet described in published literature, software must be made available to editors/reviewers. We strongly encourage code deposition in a community repository (e.g. GitHub). See the Nature Research guidelines for submitting code & software for further information.

Data

Policy information about availability of data

All manuscripts must include a data availability statement. This statement should provide the following information, where applicable:

- Accession codes, unique identifiers, or web links for publicly available datasets
- A list of figures that have associated raw data
- A description of any restrictions on data availability

Data supporting the findings of this manuscript are available from the corresponding author upon reasonable request. A reporting summary for this article is available as a Supplementary Information file.
The source data underlying Figs. 2b-c, 3c-d, 4b-h, 5b-c, 6b-d, 6f-i and Supplementary Figs 9d, 10a, 10c-f, 11b-c, 12, 13a, 14, 15a-c, 16a-e, 18a-b, 20c-e, 21a-m are provided as a Source Data file.

Field-specific reporting

Please select the one below that is the best fit for your research. If you are not sure, read the appropriate sections before making your selection.

- [x] Life sciences
- [ ] Behavioural & social sciences
- [ ] Ecological, evolutionary & environmental sciences

For a reference copy of the document with all sections, see nature.com/documents/nr-reporting-summary-flat.pdf

Life sciences study design

All studies must disclose on these points even when the disclosure is negative.

Sample size	Described in figure legends
Data exclusions	N/A
Replication	N/A
Randomization	N/A
Blinding	N/A

Reporting for specific materials, systems and methods

We require information from authors about some types of materials, experimental systems and methods used in many studies. Here, indicate whether each material, system or method listed is relevant to your study. If you are not sure if a list item applies to your research, read the appropriate section before selecting a response.

Materials & experimental systems

n/a	Involved in the study	
☐	☒	Antibodies
☐	☒	Eukaryotic cell lines
☒	☐	Palaeontology
☒	☐	Animals and other organisms
☒	☐	Human research participants
☒	☐	Clinical data

Methods

n/a	Involved in the study	
☒	☐	ChIP-seq
☐	☒	Flow cytometry
☒	☐	MRI-based neuroimaging

Antibodies

Antibodies used	All the catalog numbers are provided in the Materials section.
Validation	Validated by the supplier.

Eukaryotic cell lines

Policy information about cell lines

Cell line source(s)	Yes
Authentication	No
Mycoplasma contamination	Yes, it was regularly checked. See : Methods section / Cell culture paragraph
Commonly misidentified lines (See ICLAC register)	Yes

Flow Cytometry

Plots

Confirm that:

- [x] The axis labels state the marker and fluorochrome used (e.g. CD4-FITC).
- [x] The axis scales are clearly visible. Include numbers along axes only for bottom left plot of group (a 'group' is an analysis of identical markers).
- [x] All plots are contour plots with outliers or pseudocolor plots.
- [x] A numerical value for number of cells or percentage (with statistics) is provided.

Methodology

Sample preparation	see Methods
Instrument	BD FACSDiva for cell sorting
Software	BD FACSDiva
Cell population abundance	see Methods and Supplementary Fig 9
Gating strategy	see Supplementary Fig 9

- [ ] Tick this box to confirm that a figure exemplifying the gating strategy is provided in the Supplementary Information.
